# TACE inhibition: a promising therapeutic intervention against AATF‐mediated steatohepatitis to hepatocarcinogenesis

**DOI:** 10.1002/1878-0261.13646

**Published:** 2024-04-01

**Authors:** Akshatha N. Srinivas, Diwakar Suresh, Prashant M. Vishwanath, Suchitha Satish, Prasanna K. Santhekadur, Saisudha Koka, Divya P. Kumar

**Affiliations:** ^1^ Department of Biochemistry, CEMR Lab, JSS Medical College JSS Academy of Higher Education and Research Mysuru India; ^2^ Department of Pathology, JSS Medical College and Hospital JSS Academy of Higher Education and Research Mysuru India; ^3^ Department of Pharmaceutical Sciences, Irma Lerma Rangel School of Pharmacy Texas A&M University Kingsville TX USA

**Keywords:** Apoptosis antagonizing transcription factor, hepatocellular carcinoma, Marimastat, metabolic dysfunction associated steatohepatitis, tumor necrosis factor (TNF)‐alpha converting enzyme

## Abstract

Metabolic dysfunction‐associated steatohepatitis‐driven hepatocellular carcinoma (MASH‐HCC) is a global clinical challenge for which there is a limited understanding of disease pathogenesis and a subsequent lack of therapeutic interventions. We previously identified that tumor necrosis factor‐alpha (TNF‐α) upregulated apoptosis antagonizing transcription factor (AATF) in MASH. Here, we investigated the effect of TNF‐α converting enzyme (TACE) inhibition as a promising targeted therapy against AATF‐mediated steatohepatitis to hepatocarcinogenesis. A preclinical murine model that recapitulates human MASH‐HCC was used in the study. C57Bl/6 mice were fed with chow diet normal water (CD) or western diet sugar water (WD) along with a low dose of carbon tetrachloride (CCl_4_; 0.2 μL·g^−1^, weekly) for 24 weeks. TACE activity, TNF‐α levels, and *AATF* expression were measured. The mice were treated with the TACE inhibitor Marimastat for 12 weeks, followed by analyses of liver injury, fibrosis, inflammation, and oncogenic signaling. *In vitro* experiments using stable clones of AATF control and AATF knockdown were also conducted. We found that *AATF* expression was upregulated in WD/CCl_4_ mice, which developed severe MASH at 12 weeks and advanced fibrosis with HCC at 24 weeks. WD/CCl_4_ mice showed increased TACE activity with reduced hepatic expression of sirtuin 1 (*Sirt1*) and tissue inhibitor of metalloproteinase 3 (*Timp3*). The involvement of the SIRT1/TIMP3/TACE axis was confirmed by the release of TNF‐α, which upregulated AATF, a key molecular driver of MASH‐HCC. Interestingly, TACE inhibition by Marimastat reduced liver injury, dyslipidemia, *AATF* expression, and oncogenic signaling, effectively preventing hepatocarcinogenesis. Furthermore, Marimastat inhibited the activation of JNK, ERK1/2, and AKT, which are key regulators of tumorigenesis in WD/CCl_4_ mice and in *AATF* control cells, but had no effect on *AATF* knockdown cells. This study shows that TACE inhibition prevents AATF‐mediated inflammation, fibrosis, and oncogenesis in MASH‐HCC, offering a potential target for therapeutic intervention.

## Graphical abstract

The SIRT1/TIMP3/TACE axis is involved in the release of TNF‐α, which upregulates AATF, a key molecular driver of MASH‐HCC. This study shows that inhibition of TNF‐α converting enzyme (TACE) by Marimastat reduces liver injury, dyslipidemia, AATF expression, and oncogenic signaling, effectively preventing hepatocarcinogenesis. Thus, TACE inhibition represents a promising targeted therapy against AATF‐mediated steatohepatitis and hepatocarcinogenesis.

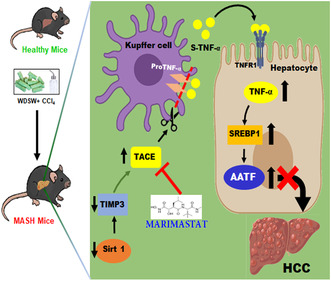

**Abbreviations**
AATF: apoptosis antagonizing transcription factor
ADAM17: A disintegrin and metalloprotease 17
AKT: protein kinase B
ALT: alanine aminotransferase
AST: aspartate aminotransferase
CCl_4_
: carbon tetrachloride
CD: chow diet normal water
CD31: cluster of differentiation 31
CHOP: C/EBP homologous protein
col1A1: collagen 1 alpha 1
col3a1: collagen 3 alpha 1
ERK1/2: extracellular signal‐regulated kinase 1/2
Grp78: 78 kDa glucose‐regulated protein
HCC: hepatocellular carcinoma
HOMA‐IR: homeostatic model assessment of insulin resistance
IL‐1β: interleukin‐1β
IL‐6: interleukin‐6
JNK: c‐Jun N‐terminal kinases
MASH: metabolic dysfunction‐associated steatohepatitis
MASLD: metabolic dysfunction‐associated steatotic liver disease
MMPs: matrix metalloproteinases
NAFLD: nonalcoholic fatty liver disease
NASH: nonalcoholic steatohepatitis
SIRT1: silent information regulator 1
SREBP1: sterol regulatory element‐binding protein 1
STAT3: signal transducer and activator of transcription 3
TACE: TNF‐alpha converting enzyme
TGF β: transforming growth factor β
TIMP3: tissue inhibitor of metalloproteinase 3
TNFR: tumor necrosis factor‐alpha receptor
TNF‐α: tumor necrosis factor‐alpha
WD: western diet sugar water
αSMA: alpha smooth muscle actin

## 1 Introduction

Metabolic dysfunction‐associated steatotic liver disease (MASLD), formerly known as nonalcoholic fatty liver disease (NAFLD), has emerged as a leading cause of chronic liver disease worldwide, with metabolic dysfunction‐associated steatohepatitis (MASH, formerly NASH) representing the more severe form [1]. Metabolic dysfunction‐associated steatohepatitis is characterized by hepatic steatosis, inflammation, and hepatocellular injury, making it a significant concern due to its potential progression to advanced liver‐related complications [2]. Among these complications, hepatocellular carcinoma (HCC) stands out as a major clinical challenge. Metabolic dysfunction‐associated steatohepatitis is gaining a greater prominence on a global scale, and it is acknowledged as the leading contributor to HCC in both developed and developing nations [3]. Of note, while the occurrence of HCC specifically linked to MASH without cirrhosis accounts for 8% of all HCC cases, the incidence rate of HCC among individuals with MASH and cirrhosis ranges from 2% to 13% [4]. Chronic inflammation and the resulting oxidative stress, compounded by genetic alterations and the progression of liver fibrosis, collectively create an environment conducive to tumorigenesis [5]. Moreover, insulin resistance and perturbations in lipid metabolism further contribute to abnormal cell growth and survival. Immune system dysregulation and shifts in the gut microbiota hinder the body's natural defenses against cancer [6]. On a cellular level, genetic and epigenetic events orchestrate a pro‐oncogenic milieu against a background of insulin resistance and inflammation [7, 8]. This progression from MASH to HCC occurs gradually, influenced by a myriad of factors, underscoring the critical importance of early intervention and continuous monitoring to manage the risk of HCC in individuals with MASH. Albeit, the link between chronic liver inflammation and hepatocarcinogenesis is well‐established, the specific mechanisms underlying MASH‐associated HCC development remain poorly understood. In recent times, studies have reported an alarming increase in the incidence of HCC in the context of MASH, surpassing that of viral hepatitis‐related HCC [9]. This epidemiological trend emphasizes the urgent need for a better understanding of the pathogenesis of MASH‐associated HCC.

Chronic inflammation plays a crucial role in MASH by creating a microenvironment that promotes cellular damage, fibrosis, and the eventual promotion of malignant transformation [10, 11]. Tumor necrosis factor‐alpha (TNF‐α), a proinflammatory cytokine involved in the pathogenesis of MASH, exerts its effects by binding to specific receptors, initiating a cascade of signaling events that lead to inflammation, immune cell activation, and tissue remodeling [12]. The availability and activity of TNF‐α in the body are tightly regulated by TNF‐alpha converting enzyme (TACE). TNF‐alpha converting enzyme, also known as a disintegrin and metalloproteinase 17 (ADAM17), is an enzyme responsible for the cleavage and release of TNF‐α from its membrane‐bound form. The soluble TNF‐α can then bind to its receptors, TNF receptor 1 (TNFR1) and TNF receptor 2 (TNFR2), initiating downstream signaling pathways to exert systemic effects [13]. Notably, increased TACE activity upon metabolic stress is shown to induce insulin resistance and hepatosteatosis [14, 15]. Thus, the strategies targeting TACE have been considered effective in treating MASLD.

We for the first time elucidated the role of apoptosis antagonizing transcription factor (AATF) and identified the mechanism involving TNF‐α/SREBP1 that leads to the upregulation of AATF, a potential driver of HCC in MASLD [16]. Apoptosis antagonizing transcription factor has been implicated in breast cancer, lung cancer, colon cancer, osteosarcoma, and neuroblastoma and is known to promote oncogenesis through diverse mechanisms, including upregulating antiapoptotic and chemokine genes, enhancing cell survival, and suppressing proapoptotic genes [17, 18, 19, 20, 21]. We recently provided evidence that AATF inhibition exerts anti‐angiogenic effect in HCC and that targeting AATF can be an effective therapeutic strategy in the treatment of HCC [22]. Given the vital role of AATF in cellular homeostasis and embryogenesis, it is important to note that the global knockout of AATF is lethal, highlighting its significance in normal physiological processes [23]. Furthermore, there are currently no approved inhibitors specifically designed to target AATF. In the light of these challenges, we hypothesized that targeting an upstream molecule in the TNF‐α/SREBP1 pathway could offer a potential therapeutic strategy for AATF mediated MASH‐HCC.

In this study, we conducted systematic research using murine and cellular models to examine whether the prevention of AATF‐mediated progression from MASH to HCC could be achieved by inhibiting TACE activity. In order to accomplish this objective, we demonstrated increased expression of AATF in the experimental MASH‐HCC model, a well‐defined mouse model that replicates the progression of the disease, starting from steatohepatitis and progressing to fibrosis and HCC. This model closely mirrors the metabolic, histological, and transcriptomic features observed in human MASH‐HCC [24]. We also investigated the impact of molecular interventions targeting the TACE pathway on MASH‐HCC evolution by using Marimastat, a pharmacological inhibitor of TACE. Thus, we provide proof of principle that targeting TACE activity could offer potential solutions for the prevention of the AATF‐mediated progression of steatohepatitis to hepatocarcinogenesis.

## 2 Materials and methods

### 2.1 Materials

The western diet was customized and purchased from Research Diets Inc. (New Brunswick, NJ, USA). The ALT, AST, and total cholesterol kits were from Monlab tests (Cornellà de Llobregat, Barcelona, Spain); Marimastat was procured from Cayman Chemicals (Ann Arbor, MI, USA), AATF antibody, carboxy methyl cellulose (CMC), corn oil, TRIzol, Sirius red stain, and RIPA buffers were from Sigma‐Aldrich (St. Louis, MO, USA). FBS, glutamine, and Lipofectamine 3000 were from Invitrogen (Waltham, MA, USA); verso cDNA synthesis kit, and DyNamo Colorflash SYBR green kit from Thermo Fisher Scientific (Waltham, MA, USA); western blotting materials were from Bio‐Rad (Hercules, CA, USA) and western bright ECL HRP substrate from Advansta (San Jose, CA, USA). Glucose, fructose, DMSO, Dulbecco's modified eagle's medium (DMEM), and hematoxylin were procured from HiMedia Laboratories (Thane, Maharashtra, India); primers from Integrated DNA Technologies (IDT) (Coralville, IA, USA), antibodies to pAKT, pJNK, pERK1/2, AKT, JNK, ERK1/2, and control and AATF shRNA from Santa Cruz Biotechnology, Inc. (Dallas, TX, USA); antibodies to Ki67, pSTAT3, STAT3, desmin and β‐actin from Cell Signaling Technologies (Danvers, MA, USA).

### 2.2 Mouse model and study design

Male C57Bl/6NCrl mice aged 6–7 weeks were obtained from Hylasco Biotechnology Pvt. Ltd. (Charles River Laboratories, Hyderabad, Telangana, India). The mice were housed in the animal facility of the Center for Experimental Pharmacology and Toxicology, JSS AHER, under a 12‐h light and 12‐h dark cycle as described previously [21]. The mice were acclimatized for 1 week before the start of the study. All procedures involving the animals were conducted in accordance with the guidelines approved by the Committee for the Purpose of Control and Supervision of Experiments on Animals (CPCSEA), Govt. of India (JSSAHER/CPT/IAEC/121/2021).

- Following the acclimatization period, the mice were randomly assigned to different dietary groups. The first group was fed a chow diet along with normal water (CD). The second group received a western diet (high‐fat, high‐sucrose, and high‐cholesterol consisting of 21% fat, 41% sucrose, and 1.25% cholesterol by weight) along with sugar water (WD) containing 18.9 g·L^−1^ d‐glucose and 23.1 g·L^−1^ d‐fructose. Along with the dietary intervention, all mice were treated with CCl_4_ at a dose of 0.2 μL·g^−1^ of body weight dissolved in corn oil. The CCl_4_ solution was administered intraperitoneally once a week throughout the study for 24 weeks, as previously described [24].
- Another set of mice was maintained on the same diet and CCl_4_ intervention. After 12 weeks, mice were randomly divided into four groups: CD/CCl_4_ with vehicle (0.5% CMC), CD/CCl_4_ with Marimastat (100 mg·kg^−1^·day^−1^), WD/CCl_4_ with vehicle (0.5% CMC), and WD/CCl_4_ with Marimastat (100 mg·kg^−1^·day^−1^). Marimastat and the vehicle were administered daily for an additional 12 weeks via oral gavage. Throughout the studies, the mice were monitored for food intake, water intake, and body weight changes. After 24 weeks, the mice were euthanized, and blood and tissues were collected for further analysis.

### 2.3 Blood collection and serum analysis

Blood samples were collected from the overnight fasted mice using the retro‐orbital puncture. The collected blood samples were centrifuged at 1500 ***g*** for 15 min at 4 °C. The serum samples were collected and stored at −80 °C until further analysis. Serum levels of alanine aminotransferase (ALT), aspartate aminotransferase (AST), and total cholesterol were measured according to the manufacturer's instructions [25].

### 2.4 Histology of liver sections

Liver sections were fixed in 10% paraformaldehyde and embedded in paraffin. 10‐μm‐thick sections were used for staining with hematoxylin and eosin (H&E) to evaluate the severity of MASLD. Stages of fibrosis, MASLD activity score, and HCC were evaluated by an expert pathologist (blindfolded) at JSS Hospital according to the FLIP algorithm and NASH‐Clinical Research Network (CRN) criteria [26, 27].

### 2.5 Picrosirius red staining

The degree of fibrosis in liver sections was assessed by histological assessment using picrosirius red stain. Formalin‐fixed, paraffin‐embedded (FFPE) liver tissue sections were deparaffinized and rehydrated using xylene and ethanol. Hematoxylin staining was performed for 10 min to visualize nuclei, followed by Sirius red staining for 1 h to assess collagen deposition. Excess stain was removed with acidified water, and slides were mounted with coverslips. Images were captured using an Olympus BX53 microscope and quantified using the imagej software (NIH, Bethesda, MA, USA) for further analysis.

### 2.6 Immunohistochemistry

The FFPE liver sections were deparaffinized and rehydrated using xylene and gradient ethanol concentrations. Antigen retrieval was performed by incubating the sections in citrate buffer (pH 6) at 94 °C for 15 min, followed by washing with water. The sections were blocked with normal goat serum and incubated with primary antibodies (AATF, 1 : 100; Desmin, 1 : 200; Ki67, 1 : 200) overnight at 4 °C. The sections were then incubated with the polyexcel HRP/DAB detection system‐one step (PathnSitu, Biotechnologies, Securderbad, Telangana, India) to develop signals and counterstained with hematoxylin. The slides were visualized with the Olympus BX53 microscope and quantified using the imagej software.

### 2.7 Measurement of TACE activity

TACE activity was determined using the SensoLyte 520 TACE Activity Assay Kit (AnaSpec, Fremont, CA, USA) according to the manufacturer's protocol. 30 μg tissue proteins or 30 μg cell proteins were used for the assay. A reaction was started by adding 40 μm of the fluorophore QXL520/5FAM FRET substrate. After incubation for 30 min, the fluorescence of the cleavage product was measured in a fluorescence microplate reader (EnSpire™ Multimode Plate Reader, Perkin Elmer, Waltham, MA, USA) at excitation/emission 490/520 nm.

### 2.8 Enzyme‐linked immunosorbent assay (ELISA)

The concentrations of fasting insulin and TNF‐α in mice serum and TNF‐α levels in cells were measured using the ELISA kits (Krishgen Biosystems, Mumbai, Maharashtra, India) according to the manufacturer's protocol.

### 2.9 RNA isolation and qRT‐PCR analysis

Total RNA was isolated from frozen liver tissue and cells using TRIzol. The concentration and purity of RNA were determined using a Nanodrop spectrophotometer. The RNA was reverse‐transcribed into cDNA using a Verso cDNA synthesis kit according to the manufacturer's instructions. Quantitative real‐time PCR was carried out using the DyNamo Colorflash SYBR green kit in the Rotor‐Gene Q 5plex HRM system (QIAGEN, Hilden, Germany). 0.5 mm primer and 50 ng of cDNA were used in a 20 μL reaction volume. The relative fold change in mRNA levels was calculated as $2^{-\Delta \Delta C_{t}}$ and normalized with β‐actin, endogenous control. The validated primer sequences used in the study are provided in Table S1.

### 2.10 Western blot analysis

Liver tissue and cell lysates were prepared using RIPA buffer containing protease inhibitors. Tissue homogenate was centrifuged at 15 870 ***g*** for 10 min at 4 °C. The supernatant was collected and subjected to protein estimation by Bradford's protein estimation method. SDS/PAGE was carried out by loading an equal amount of lysate (50 μg) to separate the protein. Further proteins are transferred to a nitrocellulose membrane. 5% skim milk was used to block the membrane for 1 h, followed by incubation with primary antibodies overnight at 4 °C. The next day, membranes were probed with a secondary antibody for 2 h. Blots were visualized using western bright ECL HRP substrate in the UVitec Alliance Q9 chemiluminescence imaging system. Images were analyzed using the imagej software, and bands were normalized with corresponding internal controls.

### 2.11 Cell culture and stable transfection

The human HCC cell line, QGY‐7703 (RRID: CVCL_6715; kindly donated by Dr Devanand Sarkar, Virginia Commonwealth University, USA) was cultured in Dulbecco's modified Eagle's medium (DMEM) supplemented with 4.5 g·L^−1^ glucose, 10% fetal bovine serum, l‐glutamine, and 100 U·mL^−1^ penicillin–streptomycin. The cells were incubated at 37 °C in a 5% CO_2_ humidified atmosphere. The authenticity of QGY‐7703 cells was confirmed by short tandem repeat (STR) profiling and was tested for mycoplasma contamination.

Stable clones expressing AATF shRNA in QGY‐7703 cells were generated as described previously [12]. To determine the optimal antibiotic concentration for selecting stable cell colonies, different concentrations of puromycin (ranging from 1 to 10 μg·mL^−1^) were used. The control and AATF shRNA plasmids, which contained the puromycin resistance gene, were transfected into QGY‐7703 cells following the manufacturer's instructions. Single colonies were selected and subsequently cultured in a medium containing 1 μg·mL^−1^ puromycin. The knockdown of AATF was confirmed through both qRT‐PCR and western blotting analysis.

### 2.12 Statistical analysis

Results were calculated as the mean ± SEM. Statistical significance was analyzed using the Student's *t*‐test for two groups or analysis of variance (ANOVA) with *post hoc* Bonferroni correction for multiple comparisons. All statistical analyses were performed using the graphpad prism software (Dotmatics, Boston, MAA, USA) (version 6), and *P* values < 0.05 (*/#) or < 0.001 (**/##) were considered significant.

## 3 Results

### 3.1 AATF is overexpressed in experimental HCC driven by MASH

**Fig. 1:**
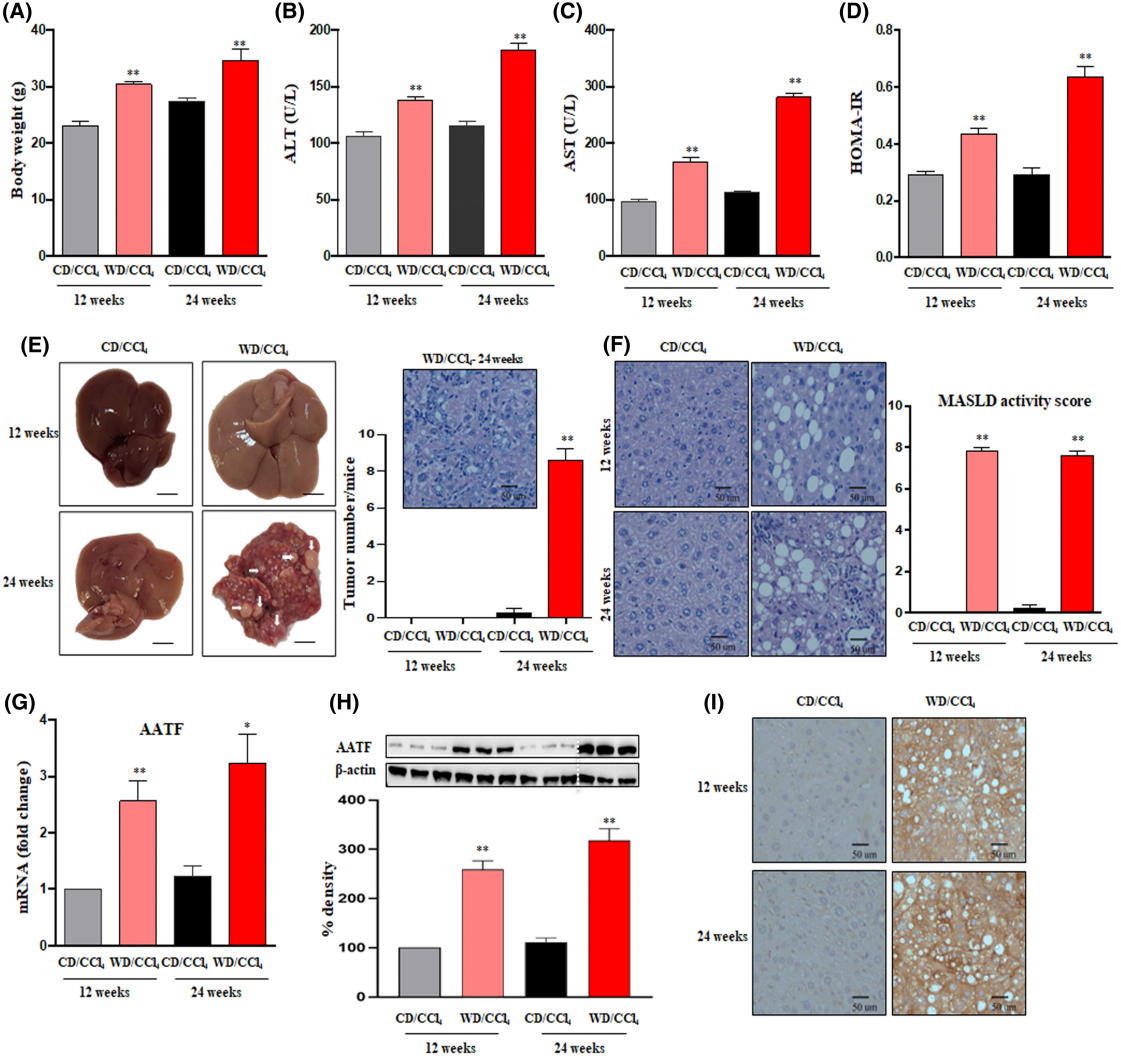
Increased hepatic AATF expression in the progressive stages of MASLD. Mice were fed with either CD/CCl_4_ or WD/CCl_4_ for 12 and 24 weeks. After the completion of treatment, body weight (A), serum ALT (B), and serum AST (C) were estimated. (D) HOMA‐IR was calculated using fasting insulin and fasting glucose values using the standard formula: Fasting glucose (mg·dL^−1^) X fasting insulin (mU·L^−1^)/405. (E) Representative liver images of CD/CCl_4_ or WD/CCl_4_ mice at 12 and 24 weeks with histology and tumor burden. Arrows represent the tumor nodules. (F) Representative microscopic images of H&E‐stained liver sections depicting steatosis, hepatocyte ballooning, and lobular inflammation with MASLD activity score. Hepatic AATF expression as measured by qRT‐PCR and expression normalized to β‐Actin (G), western blot (68 kDa) (*n* = 3 mice per group) (H), and immunohistochemistry (I). The dotted white line in the western blot (H) indicates the vertically spliced images that are juxtaposed lanes from non‐adjacent gels. All the samples were derived from the same experiment and the blots were processed in parallel. Loading control (β Actin) is run on the same blot (*n* = 3 independent experiments). For all images, magnification: 400× And scale bar: 50 μm. Statistical significance was analyzed by a Student's *t*‐test. Data expressed as mean ± SEM for *n* = 6 mice per group except for (H). **P < 0.001 or *P < 0.05 compared to CD/CCl4. AATF, apoptosis antagonizing transcription factor; ALT, alanine transaminase; AST, aspartate aminotransferase; CCl_4_, carbon tetrachloride; CD, chow diet; HOMA‐IR, homeostatic model assessment for insulin resistance; MASLD, metabolic dysfunction associated steatotic liver disease; WD, western diet.

To evaluate the molecular mechanisms underlying inflammation and carcinogenesis in MASH‐associated HCC, C57Bl/6 mice were fed with either chow diet normal water (CD) or western diet sugar water (WD) along with a weekly intraperitoneal injection of low‐dose CCl_4_ (0.2 μL·g^−1^ body weight, which is substantially less than the amount typically used to induce fibrosis with CCl_4_ alone) for 24 weeks. Mice were euthanized at 12 and 24 weeks to study the metabolic and histological features of MASH and HCC, respectively. In contrast to CD/CCl_4_ mice, there was a significant increase in the body weight and liver weight of WD/CCl_4_ mice at 12 and 24 weeks (Fig. 1A and Fig. S1A). Elevated serum AST and ALT levels marked the occurrence of underlying liver injury in WD/CCl_4_ mice at 12 and 24 weeks (Fig. 1B,C). Similarly, metabolic parameters such as serum total cholesterol, fasting blood glucose, and fasting insulin levels were significantly higher in WD/CCl_4_ mice compared to CD/CCl_4_ mice (Fig. S1B–D). Insulin resistance, marked by HOMA‐IR measurement, was also found to be increased at 12 and 24 weeks (Fig. 1D). Importantly, throughout the duration of the study, there was consistent stability in caloric and food intake across all four groups.

Consistent with the biochemical and metabolic parameters, administration of WD/CCl_4_ developed MASH at 12 weeks and HCC at 24 weeks (Fig. 1E). Histological examination showed significant micro‐ and macro‐vesicular steatosis in WD/CCl_4_ mice at 12 weeks and multinucleate, poorly differentiated tumor morphology along with steatosis at 24 weeks (Fig. 1E,F). Furthermore, WD/CCl_4_ mice showed severe steatosis at 12 weeks (2.5 ± 0.22) and 24 (2.8 ± 0.16) weeks followed by significant lobular inflammation (12 weeks: 2.2 ± 0.2; 24 weeks: 2.2 ± 0.3) and hepatocyte ballooning (12 weeks: 1.6 ± 0.2; 24 weeks: 1.7 ± 0.2) (Fig. S1E–G). The severity of the disease characterized by the MASLD activity score in WD/CCl_4_ mice was 7.8 ± 0.2 at 12 weeks and 7.4 ± 0.31 at 24 weeks.

To confirm the role of AATF as evidenced by our previous studies, we measured the expression of AATF both at mRNA and protein levels in the mouse model of HCC driven by MASH. AATF was found to be overexpressed both at mRNA and protein levels in the liver tissues of WD/CCl_4_ mice at 12 and 24 weeks compared with CD/CCl_4_ mice (Fig. 1G–I).

### 3.2 Activation of inflammation, ER stress, fibrosis, and oncogenic signaling during MASH to HCC

**Fig. 2:**
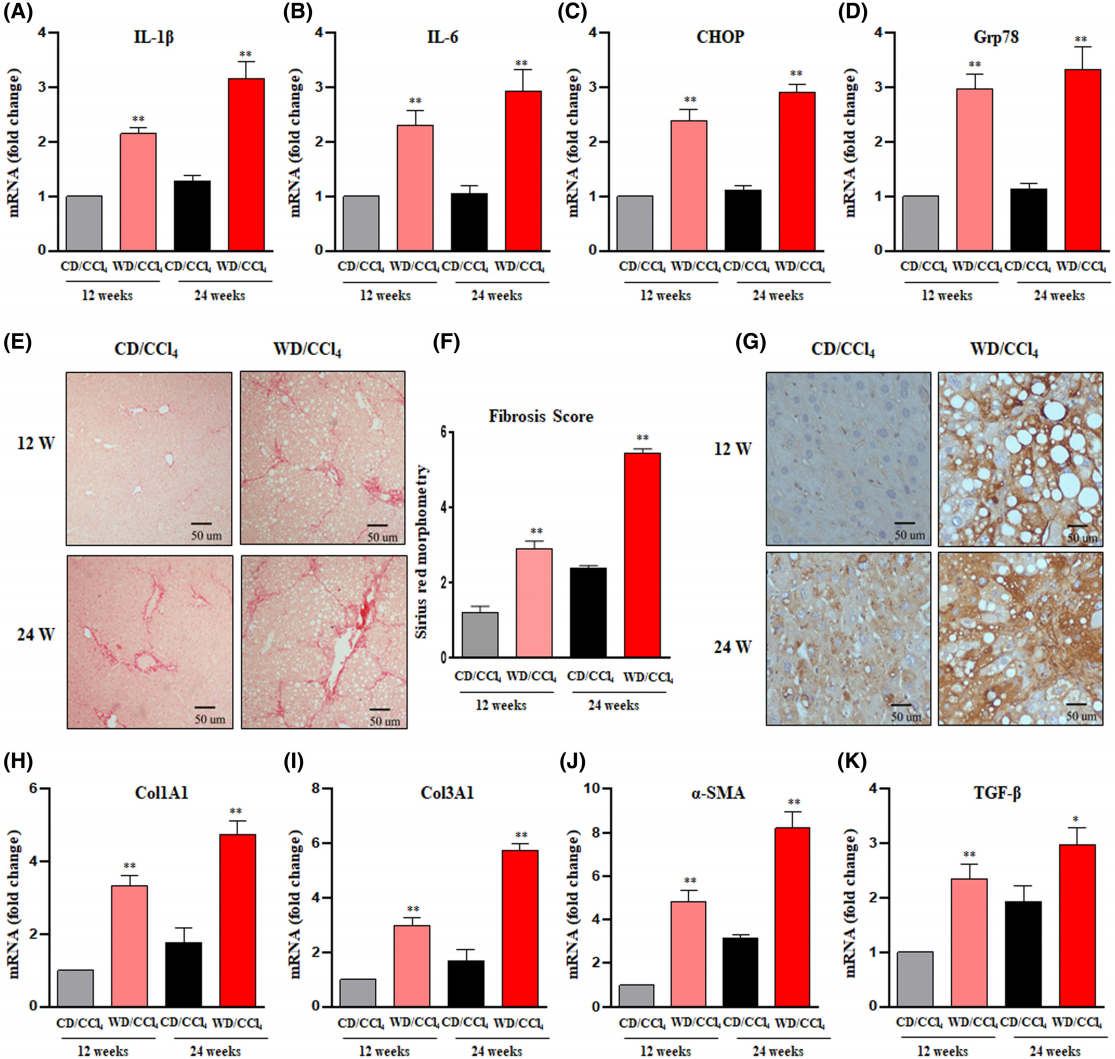
Evaluation of inflammatory cytokines, ER stress, and fibrosis markers in CD/CCl_4_ and WD/CCl_4_ mice. The relative hepatic mRNA expression of (A) IL‐1β, (B) IL‐6, (C) CHOP, and (D) Grp78 was estimated by qRT‐PCR and normalized to the endogenous control, β‐Actin. (E) Representative microscopic images of picrosirius red staining with fibrosis score (F) of mice treated with CD/CCl_4_ and WD/CCl_4_ for 12 and 24 weeks. (G) Immunostaining of desmin done in liver sections, and the relative hepatic mRNA expression of col1A1 (H), col3a1 (I), α‐SMA (J), and TGF β (K) as estimated by qRT‐PCR and expression normalized to the endogenous control, β‐Actin. For all images, magnification: 400× And scale bar: 50 μm. Statistical significance was analyzed by a Student's *t*‐test. Data expressed as mean ± SEM for *n* = 6 mice per group, *n* = 3 independent experiments. **P < 0.001 or *P < 0.05 compared to CD/CCl4. CCl_4_, carbon tetrachloride; CD, chow diet; CHOP, C/EBP homologous protein; col1A1, collagen 1 alpha 1; col3a1, collagen 3 alpha 1; Grp78, 78 kDa glucose‐regulated protein; IL‐1β, interleukin‐1β; IL‐6, interleukin‐6; TGF β, transforming growth factor β; WD, western diet; αSMA, alpha Smooth Muscle Actin.

The hallmarks of MASH include liver injury and chronic inflammation arising in the context of cellular stress, which subsequently trigger the activation of fibrosis and the development of cancer. Along the same lines, we examined the expression status of genes involved at various stages of the progression of MASH to HCC. WD/CCl_4_ mice showed significant inflammation characterized by elevated levels of proinflammatory cytokines, IL‐1β and IL‐6 (Fig. 2A,B). The endoplasmic reticulum (ER) stress markers CHOP and Grp78 were upregulated in WD/CCl_4_ mice at 12 and 24 weeks (Fig. 2C,D). Further, WD/CCl_4_ mice showed prominent fibrosis as demonstrated by picrosirius red staining (Fig. 2E,F) at 12 and 24 weeks compared with the CD/CCl_4_ mice. The upregulated expression of desmin and α‐SMA shows increased activation of hepatic stellate cells in WD/CCl_4_ mice at 12 and 24 weeks (Fig. 2G,J). In addition, key fibrogenic markers such as Col1a1, Col3a1, and TGF‐β were significantly upregulated in WD/CCl_4_ mice at 12 and 24 weeks (Fig. 2H–K).

**Fig. 3:**
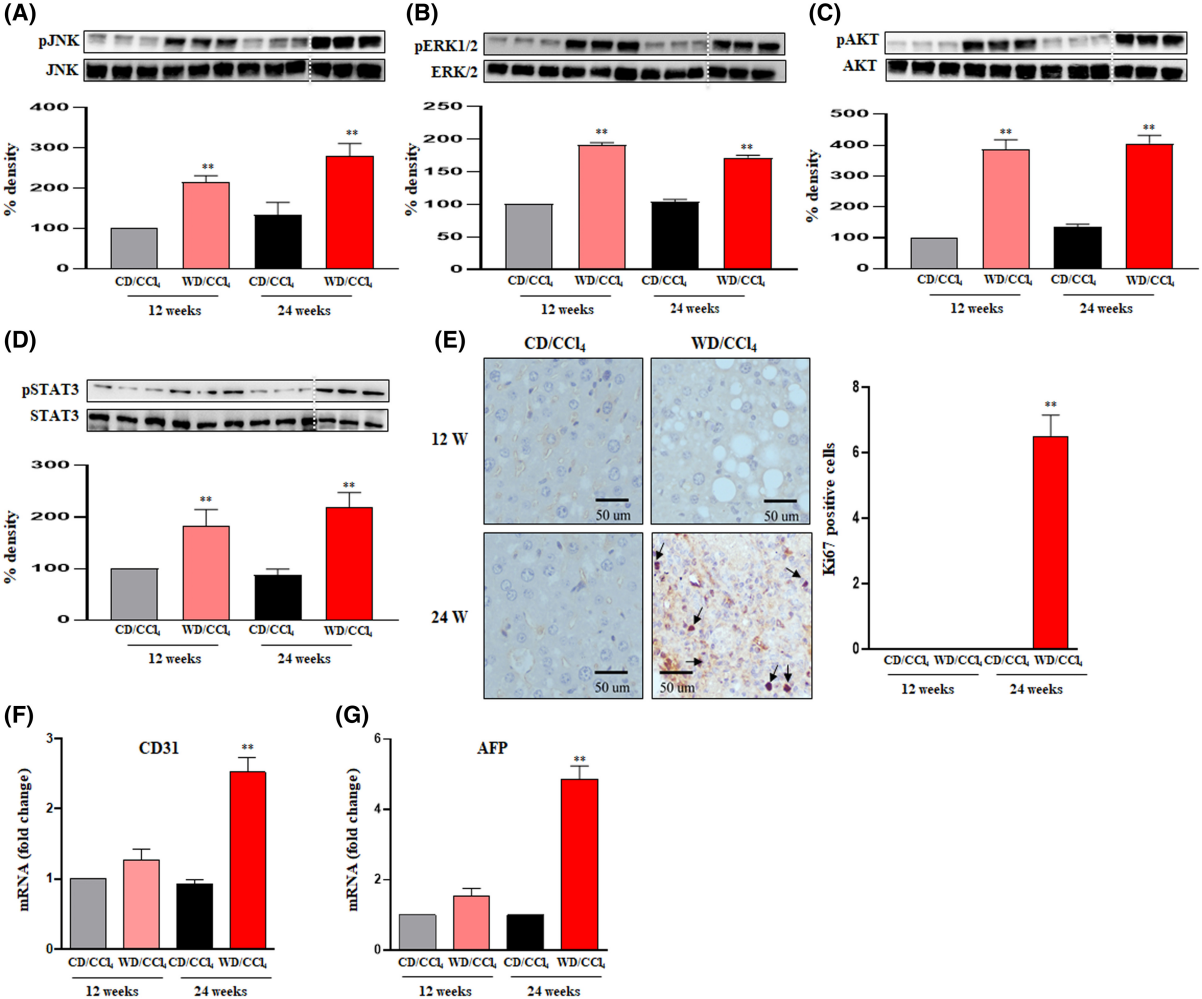
Activation of oncogenic signaling in experimental HCC driven by MASH. Western blots were performed on the whole liver tissue lysates of CD/CCl_4_ and WD/CCl_4_ mice. Representative blot images and densitometric analysis of (A) pJNK and JNK (46 kDa), (B) pERK1/2 and ERK1/2 (44 kDa), (C) pAKT and AKT (62 kDa), (D) pSTAT3 and STAT3 (86 kDa) (*n* = 3 mice per group). The dotted white line indicates the vertically spliced images that are juxtaposed lanes from non‐adjacent gels. All the samples were derived from the same experiment and the blots were processed in parallel. Loading controls are run on the same blot. (E) Representative microscopic images of Ki67 immunostaining with quantification of Ki67‐positive cells; magnification: 400× And scale bar: 50 μm. Relative hepatic mRNA expression of (F) CD31 and (G) AFP were estimated by qRT‐PCR and expression normalized to the endogenous control, β‐Actin. Statistical significance was analyzed by a Student's *t*‐test. Data expressed as mean ± SEM for *n* = 6 mice per group, *n* = 3 independent experiments **P < 0.001 compared to CD/CCl4. AFP, alpha‐fetoprotein; CCl_4_, carbon tetrachloride; CD, chow diet; CD31, cluster of differentiation 31; pAKT, phospho‐Atkin kinase; p‐ERK1/2, phospho‐extracellular signal regulated protein kinase; p‐JNK, phospho‐c‐Jun N‐terminal kinase; pSTAT3, phospho‐signal transducer and activator of transcription 3; WD, western diet.

Additionally, we also performed immunoblotting to assess the mitogen‐activated protein kinases (MAPKs), which contribute to oncogenic signaling, promoting cancer development and progression [28]. WD/CCl_4_ mice showed increased phosphorylation of extracellular signal‐regulated protein kinase (ERK1/2) and c‐Jun N‐terminal kinase (JNK). Similarly, mice treated with WD/CCl_4_ showed activation of AKT, which is closely associated with cell survival, proliferation, and migration of HCC (Fig. 3A–C). Also upregulated expression of pSTAT3, a key regulator of tumorigenesis, was observed in WD/CCl_4_ mice (Fig. 3D).

Furthermore, immunostaining of Ki67 confirmed tumorigenesis in WD/CCl_4_ mice at 24 weeks (Fig. 3E). The cluster of Differentiation 31 (CD31) level was elevated in WD/CCl_4_ mice, affirming vascular differentiation along with high expression levels of alpha‐fetoprotein (AFP), a prominent HCC marker (Fig. 3F,G). These results demonstrate that the activation of inflammation in WD/CCl_4_ mice at 12 weeks drives fibrosis and hepatocarcinogenesis, as evidenced at 24 weeks. Thus, MASH, characterized by chronic inflammation, provides a favorable setting for the transition of MASH to HCC.

### 3.3 Involvement of SIRT1/TIMP3/TACE axis in MASH‐HCC

**Fig. 4:**
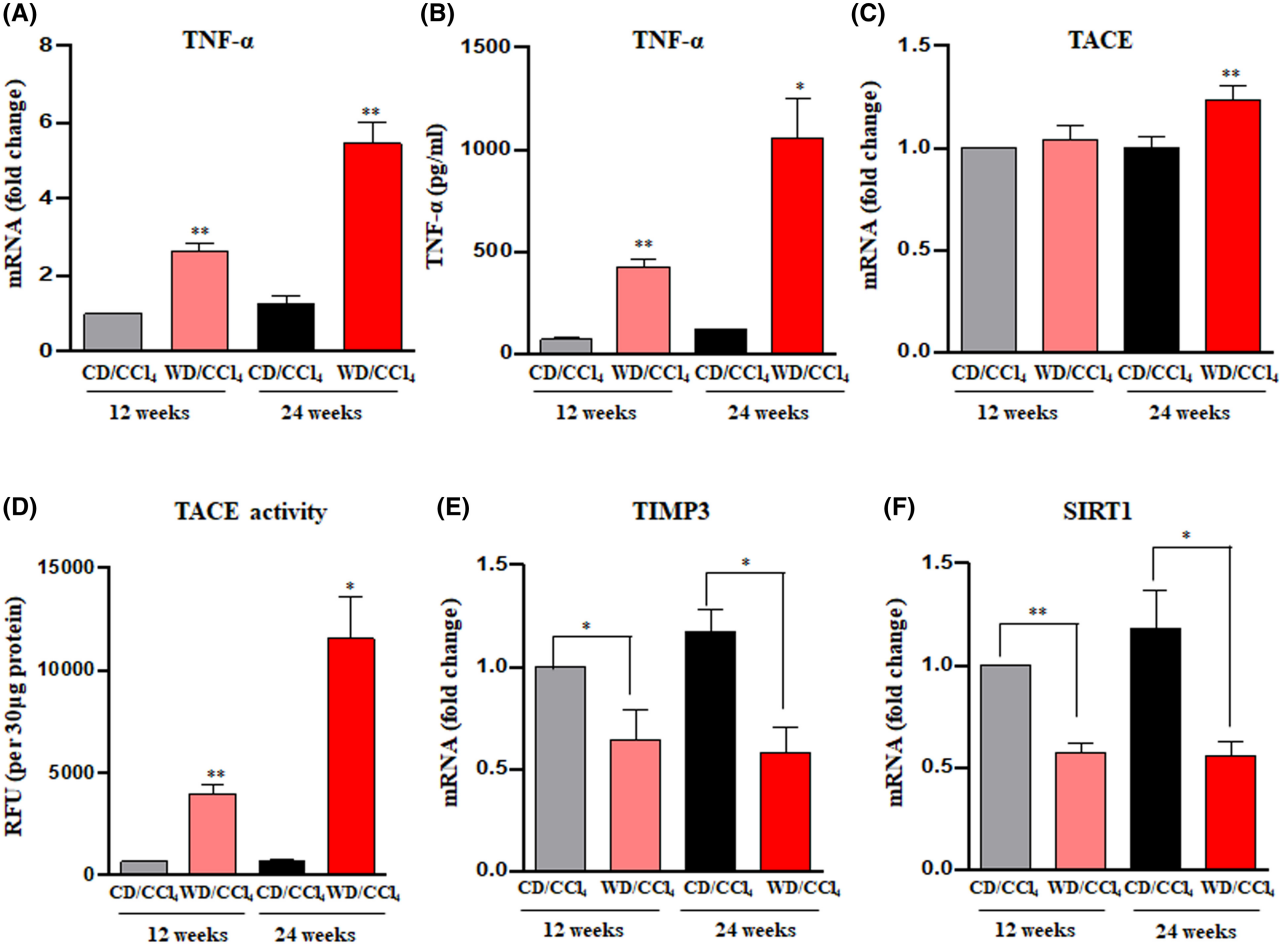
Evaluation of the role of the Sirt1/TIMP3/TACE axis in the progression of MASH to HCC. Mice were fed with either CD/CCl_4_ or WD/CCl_4_ for 12 and 24 weeks. *TNF‐α* was measured by qRT‐PCR (A) and ELISA (B). *Tace* mRNA (C) and TACE activity (D) were measured in CD/CCl_4_ or WD/CCl_4_ mice. Hepatic expression of *Timp3* (E) and *Sirt1* (F) was measured by qRT‐PCR and expression normalized to the endogenous control, β‐Actin. Statistical significance was analyzed by a Student's *t*‐test. Data expressed as mean ± SEM for *n* = 6 mice per group, *n* = 3 independent experiments. **P < 0.001 or *P < 0.05 compared to CD/CCl4. CCl_4_, carbon tetrachloride; CD, chow diet; *Sirt1*, silent information regulator 1; *Tace*, tumor necrosis factor‐alpha converting enzyme; *Timp3*, tissue inhibitor of metalloproteinase 3; TNF‐α, tumor necrosis factor‐α; WD, western diet.

Previously, we demonstrated that obesity‐induced proinflammatory cytokine (TNF‐α) upregulates AATF expression via SREBP1 [16]. In this study, we aimed to identify the upstream mechanism activating TNF‐α, a cytokine that plays a complex role in predisposing the liver to chronic inflammation and the development of cancer [29]. Mice treated with WD/CCl_4_ showed elevated levels of TNF‐α in the liver tissue and serum at 12 and 24 weeks (Fig. 4A,B), consistent with our previous studies. Given the connection between TNF‐α and TACE, which lies in the processing and release of TNF‐α from its inactive, transmembrane‐bound form to its active, soluble form [13], we examined whether TACE activity is modulated in WD/CCl_4_ mice. As hypothesized, we found that upon metabolic stress, there was increased hepatic TACE activity in mice treated with WD/CCl_4_ at12 weeks, which was further elevated at 24 weeks compared to CD/CCl_4_ mice (Fig. 4C,D).

Next, we sought to explore the inflammatory‐fibrosis‐cancer axis that promotes MASH‐associated HCC. There is a positive correlation between silent information regulator 1 (SIRT1) and tissue inhibitor of metalloproteinase 3 (TIMP‐3), which acts as a natural inhibitor of TACE [30, 31]. Of note, SIRT1 serves as a metabolic sensor and plays a vital role in the regulation of lipid and energy balance in the liver [32]. Increased levels of SIRT1 protect mice against hepatic inflammation and steatosis induced by a high‐fat diet (HFD), while its inhibition worsens these conditions [33]. Henceforth, we further validated the hepatic expression of TIMP3 and SIRT1 at 12 and 24 weeks. In contrast to the increased TACE activity, WD/CCl_4_ mice showed reduced expression of TIMP3 and SIRT1 (Fig. 4E,F). Collectively, these findings suggest that the induction of inflammation in WD/CCl_4_ mice occurs through the inhibition of SIRT1. This inhibition, in turn, leads to a decrease in TIMP3 levels (an inhibitor of TACE) and thereby subsequently enhances the activity of TACE, resulting in the release of active TNF‐α. Of note, the TCGA public database also supported our results by providing strong evidence for upregulation of AATF and TACE expression in HCC (*n* = 371) compared with normal (*n* = 50) samples with a positive correlation in their gene expression (Fig. S2).

### 3.4 TACE inhibition prevents the progression of MASH to HCC

**Fig. 5:**
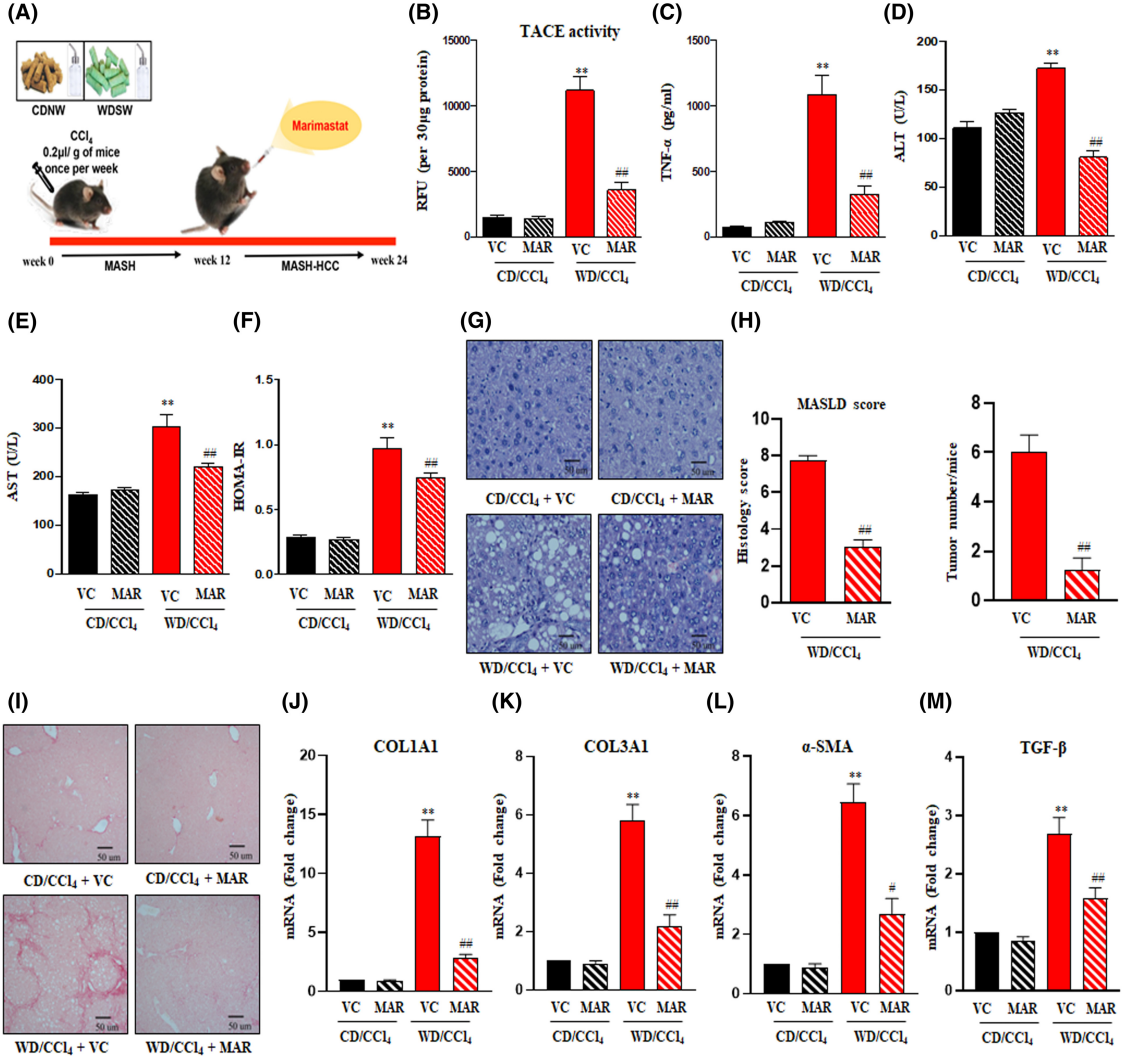
The TACE inhibitor Marimastat prevents the progression of MASH‐associated liver tumors in WD/CCl_4_ mice. (A) Study design: Mice were fed with CD/CCl_4_ or WD/CCl_4_ for 12 weeks and then treated with vehicle control or Marimastat for an additional 12 weeks. (B) TACE activity, (C) serum TNF‐α, (D) serum ALT, and (E) serum AST were measured. (F) HOMA‐IR was determined based on the values of fasting glucose and fasting insulin measurements. Representative liver histology images (G) with MASLD activity score and tumor burden (H) and microscopic images of picrosirius red staining (I) of CD/CCl_4_ or WD/CCl_4_ mice treated with or without Marimastat. For all images, magnification: 400× And scale bar: 50 μm. Hepatic mRNA expression of col1A1 (J), col3a1 (K), α‐SMA (L), and TGF β (M) as estimated by qRT‐PCR and expression normalized to the endogenous control, β‐Actin. Statistical significance was analyzed by a Student's *t*‐test. Data expressed as mean ± SEM for *n* = 6 mice per group, *n* = 3 independent experiments. **P < 0.001 to CD/CCl4 vehicle control; ^##^ *P* < 0.001 or ^#^ *P* < 0.05 compared to WD/CCl4 vehicle control. ALT, alanine transaminase; AST, aspartate aminotransferase; CCl_4_, carbon tetrachloride; CD, chow diet; col1A1, collagen 1 alpha 1; col3a1, collagen 3 alpha 1; HOMA‐IR, homeostatic model assessment for insulin resistance; MAR, Marimastat; MASH, metabolic dysfunction associated steatohepatitis; MASH‐HCC, metabolic dysfunction‐associated steatohepatitis‐induced hepatocellular carcinoma; MASLD, metabolic dysfunction associated steatotic liver disease; TACE, tumor necrosis factor‐alpha converting enzyme; TGF β, transforming growth factor β; TNF‐α, tumor necrosis factor‐α; VC, vehicle control; WD, western diet; αSMA, alpha Smooth Muscle Actin.

The TIMP3/TACE axis plays a central role in connecting the metabolic clues with the immune response, and henceforth, we explored whether any interference with this metabolic axis would affect the occurrence of HCC in the context of metabolic‐associated steatohepatitis (MASH). To answer this question, after 12 weeks of WD/CCl_4_ administration, the mice were treated with Marimastat (100 mg·kg^−1^·day^−1^), a potent TACE inhibitor, for 12 weeks (Fig. 5A). As expected, TACE activity and TNF‐α levels were reduced upon Marimastat treatment in WD/CCl_4_ when compared to vehicle control (Fig. 5B,C). Marimastat treatment for 12 weeks significantly reduced body weight and liver weight and showed improved liver injury, dyslipidemia, and HOMA‐IR in WD/CCl_4_ mice (Fig. 5D–F and Fig. S3A–F). Of note, calorie intake was not affected by the treatment with Marimastat (Fig. S3C). Consistent with these data, Marimastat had a positive effect on ameliorating steatosis (1.3 ± 0.4), hepatocellular ballooning (0.8 ± 0.27), and lobular inflammation (1.2 ± 0.38) against vehicle control mice (Fig. S3G–I). Overall MASLD activity score (4.7 ± 0.4) was significantly improved upon Marimastat treatment compared to WD/CCl_4_ mice on vehicle control (7.5 ± 0.3) (Fig. 5G).

More strikingly, there was a reduction in tumor formation upon treatment with Marimastat against severe HCC observed in vehicle control treated WD/CCl_4_ mice (Fig. 5H). We also investigated the effect of Marimastat on liver fibrosis. Picrosirius red staining showed a marked reduction in fibrosis in WD/CCl_4_ mice treated with Marimastat (Fig. 5I). Additionally, key fibrogenic markers such as Col1A1, Col3A1, α‐SMA, and TGF‐β expression levels were found to be downregulated upon Marimastat treatment (Fig. 5J–M). Of note, Marimastat treatment had no effect on CD/CCl_4_ mice. These data indicate that Marimastat, via the inhibition of TACE, suppresses the progression of MASH to HCC in WD/CCl_4_ mice.

### 3.5 Effect of TACE inhibition on AATF‐mediated MASH‐HCC

**Fig. 6:**
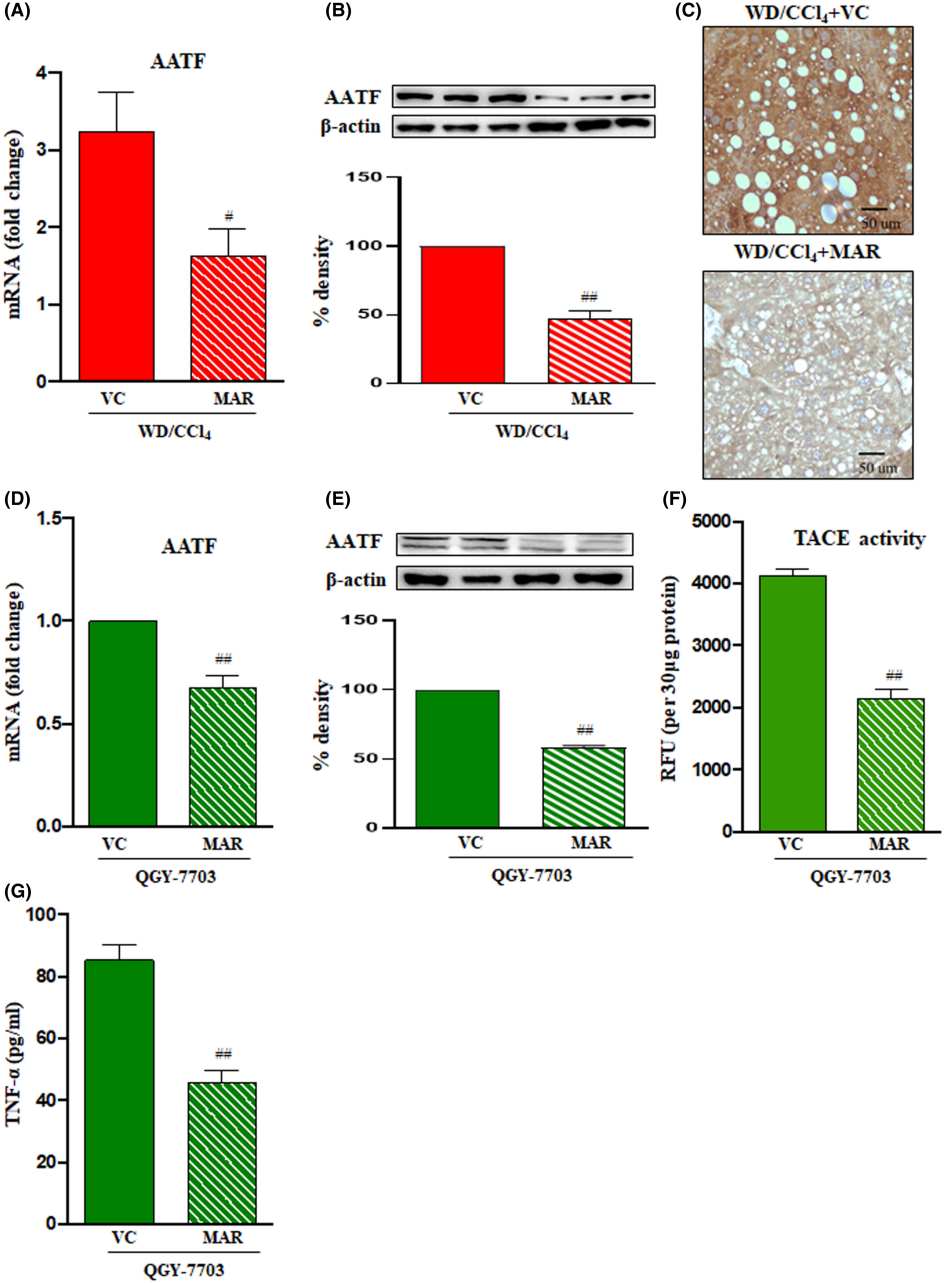
Effect of the TACE inhibitor Marimastat on AATF expression. Mice were fed with CD/CCl_4_ or WD/CCl_4_ for 12 weeks and then treated with vehicle control or Marimastat for an additional 12 weeks. *AATF* expression was measured by qRT‐PCR and expression normalized to β‐Actin (A), western blot (68 kDa; *n* = 3 mice per group) (B), and Immunohistochemistry; magnification: 400× And scale bar: 50 μm (C). Human HCC cells, QGY‐7703 cells, were treated with Marimastat, and mRNA (D) and protein (68 kDa) (E) levels of *AATF* were determined. (F) TACE activity and (G) TNF‐α were also measured in QGY‐7703 cells treated with or without Marimastat. Statistical significance was analyzed by a Student's *t*‐test. Data expressed as mean ± SEM for *n* = 6 mice per group, *n* = 3 independent experiments. ^##^ *P* < 0.001 or ^#^ *P* < 0.05 compared to WD/CCl4 or QGY‐7703 cells vehicle control. AATF, apoptosis antagonizing transcription factor; CCl_4_, carbon tetrachloride; MAR, Marimastat; TACE, tumor necrosis factor‐alpha converting enzyme; TNF‐α, tumor necrosis factor‐α; VC, vehicle control; WD, western diet.

The ability of Marimastat to suppress the transition of inflammation to cancer in WD/CCl_4_ mice prompted us to examine the effect of Marimastat on the AATF‐mediated progression of MASH to HCC. As a first step, we measured the expression of AATF upon Marimastat treatment in both mouse liver tissue and human HCC cells (the optimal concentration of Marimastat was determined for the treatment as shown in Fig. S4A; the authenticity of QGY‐7703 cells was confirmed by short tandem repeat [STR] profiling). Analysis revealed reduced expression of hepatic AATF both at the transcript and protein levels in WD/CCl_4_ mice (Fig. 6A–C). Similar results were obtained in human HCC cells, QGY‐7703, which showed reduced expression of AATF upon Marimastat treatment (Fig. 6D,E). Subsequently, the measurement of TACE activity and TNF‐α levels in QGY‐7703 cells was also reduced with Marimastat treatment (Fig. 6F,G). We also used another human HCC cell line, Hep3B, which also showed similar results in terms of TACE activity and TNF‐α levels upon Marimastat treatment (Fig. S4B,C).

**Fig. 7:**
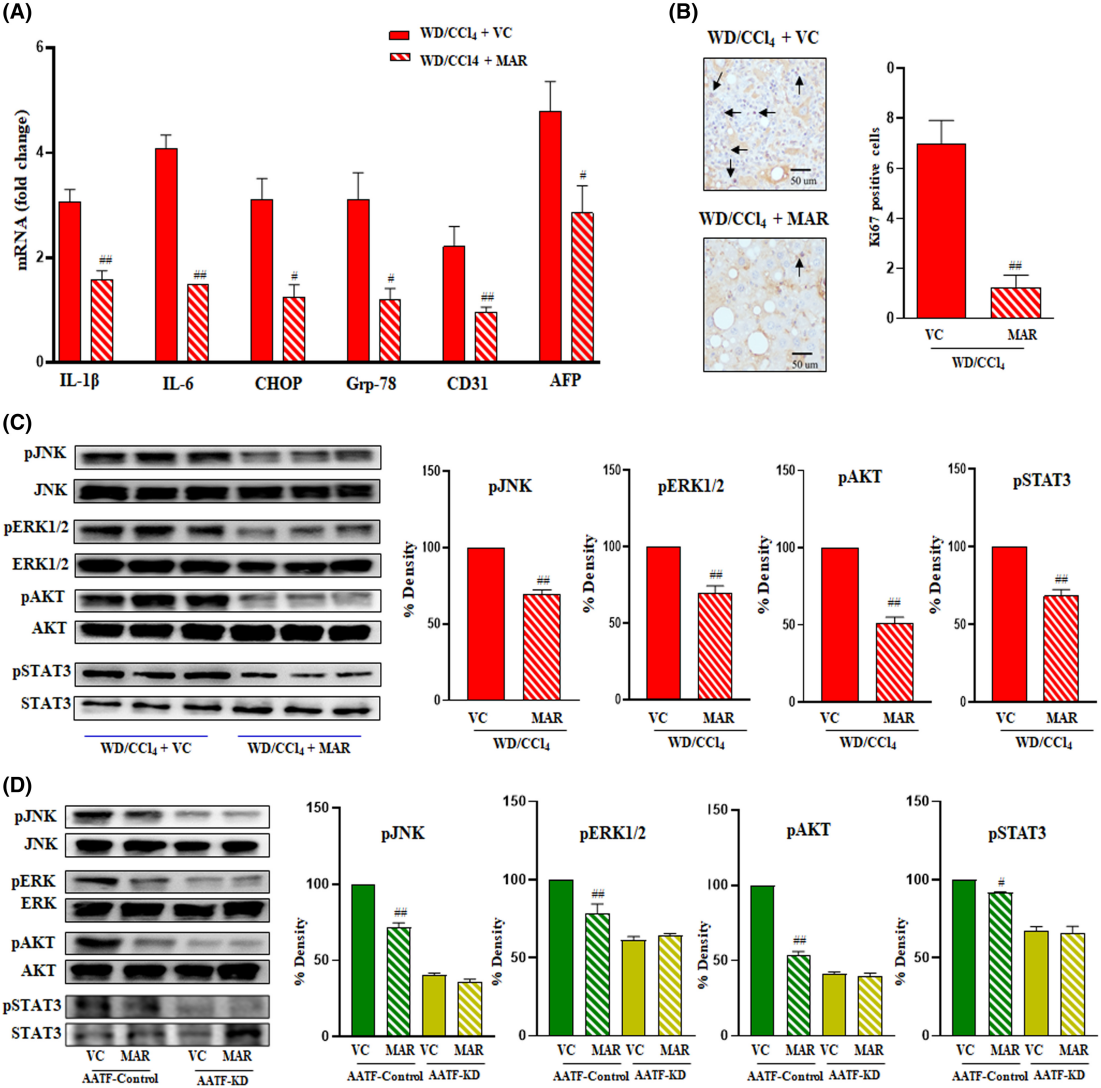
Effect of TACE inhibition on AATF‐mediated MASH‐HCC. Mice were fed with CD/CCl_4_ or WD/CCl_4_ for 12 weeks and then treated with vehicle control or Marimastat for an additional 12 weeks. (A) qRT‐PCR analysis was carried out to determine the hepatic expression of IL‐1β, IL‐6, CHOP, Grp78, CD31, and AFP. Expression normalized to β‐Actin (B) Representative microscopic images of Ki67 immunostaining with quantification of Ki67‐positive cells. Arrows represent the Ki67 positive cells; magnification: 400× And scale bar: 50 μm (C) Immunoblot images of pJNK and JNK (46 kDa), pERK1/2 and ERK1/2 (44 kDa), pAKT and AKT (62 kDa), pSTAT3 and STAT3 (86 kDa) with their respective densitometric analyses normalized to total proteins (*n* = 3 mice per group). (D) Whole cell lysates from stable clones of QGY‐7703 cells‐AATF control and AATF knockdown cells treated with or without Marimastat were subjected to immunoblot analysis of pJNK (46 kDa), pERK1/2 (44 kDa), pAKT (62 kDa), and pSTAT3 (86 kDa). Bar graphs show the densitometric values calculated after normalization to total ERK1/2, JNK, AKT, and STAT3, respectively. Statistical significance was analyzed by a Student's *t*‐test. Data expressed as mean ± SEM, *n* = 3 independent experiments. ^##^ *P* < 0.001 or ^#^ *P* < 0.05 compared to WD/CCl4 or AATF control treated with vehicle. AFP, alpha‐fetoprotein; CCl_4_, carbon tetrachloride; CD31, cluster of differentiation 31; CHOP, C/EBP homologous protein; Grp78; 78 kDa glucose‐regulated protein; IL‐1β, interleukin‐1β; IL‐6, interleukin‐6; KD, knockdown; MAR, Marimastat; pAKT, phospho‐Atkin kinase; p‐ERK1/2, phospho‐extracellular signal regulated protein kinase; p‐JNK, phospho‐c‐Jun N‐terminal kinase; pSTAT3, phospho‐signal transducer and activator of transcription 3; VC, vehicle control; WD, western diet.

**Fig. 8:**
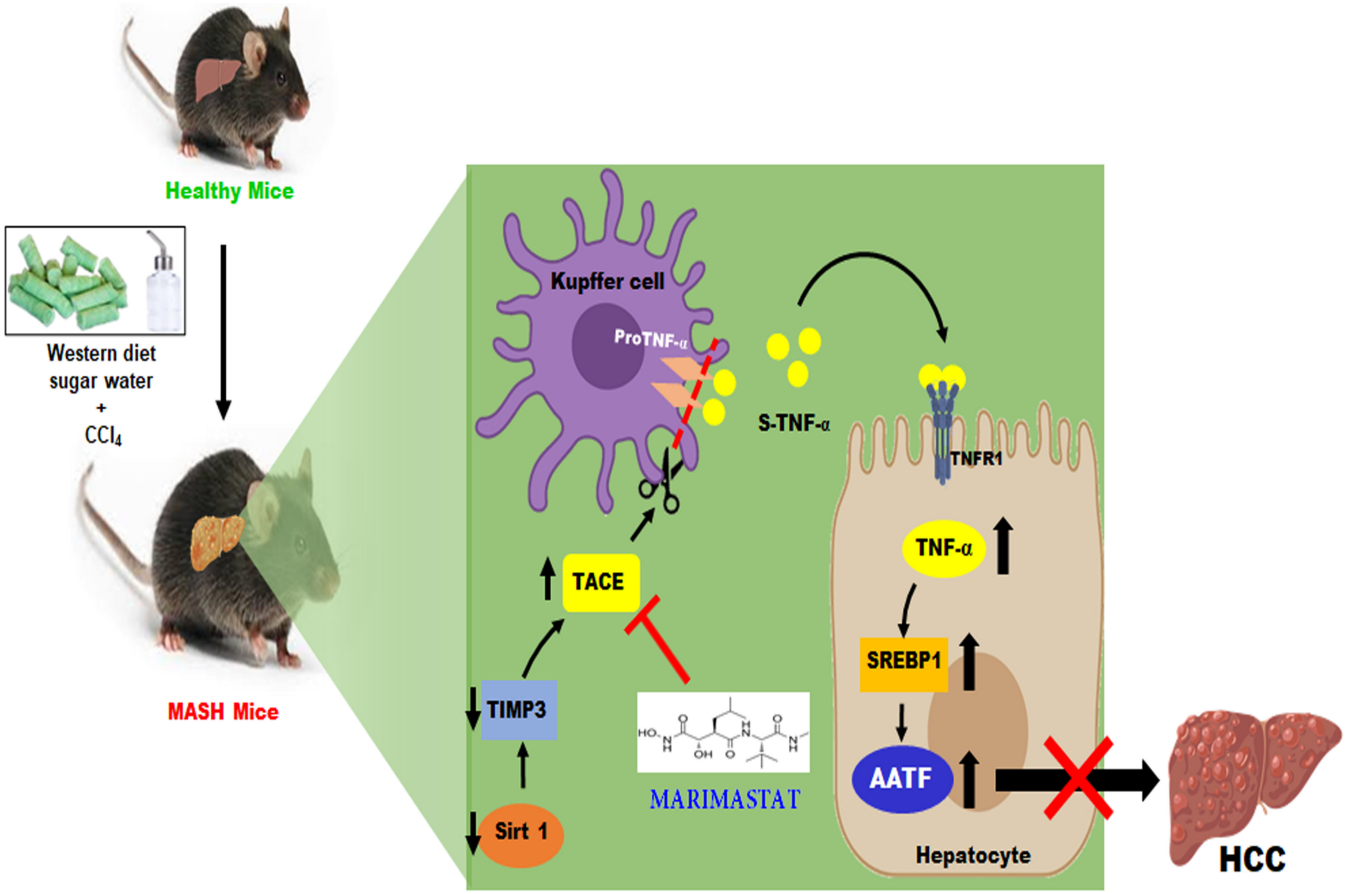
Schematic representation depicting the effect of TACE inhibition on MASH‐associated HCC mediated by AATF. Mice were fed with CD/CCl_4_ or WD/CCl_4_ for 12 weeks and then treated with vehicle control or Marimastat for an additional 12 weeks. TACE inhibition by Marimastat reduces proinflammatory cytokines and prevents the progression of MASH to HCC mediated by AATF. *AATF*, apoptosis antagonizing transcription factor; CCl_4_, carbon tetrachloride; CD, chow diet; HCC, hepatocellular carcinoma; MASH, metabolic dysfunction associated steatohepatitis; *Sirt1*, silent information regulator 1; *SREBP1*, sterol regulatory element‐binding protein 1; TACE, tumor necrosis factor‐alpha converting enzyme; *Timp3*, tissue inhibitor of metalloproteinase 3; TNFR1, tumor necrosis factor‐ α receptor 1; TNF‐α, tumor necrosis factor‐α; WD, western diet.

To better understand the AATF downregulation in WD/CCl_4_ mice treated with Marimastat, we analyzed the key signaling pathways involved in inflammation and oncogenesis. Marimastat treatment significantly reduced proinflammatory cytokines (Il‐1β and Il‐6), ER stress markers (CHOP and Grp78), and tumor markers (CD31 and AFP) (Fig. 7A). Similarly, Ki67 immunostaining was negative in the Marimastat‐treated WD/CCl_4_ mice (Fig. 7B). Immunoblot analysis of stress kinases such as pERK1/2 and pJNK, cell survival marker pAKT, and oncogenic signaling protein pSTAT3 showed marked reduction upon Marimastat treatment (Fig. 7C). Furthermore, in order to investigate the potential impact of inhibiting TACE on AATF‐driven oncogenesis, stable clones of AATF control and AATF knockdown QGY‐7703 cells were subjected to treatment with Marimastat. As anticipated, the protein concentrations of p‐JNK, p‐ERK1/2, p‐AKT, and p‐STAT3 were significantly reduced in AATF knockdown clones compared with AATF control. Likewise, in line with our hypothesis, treatment with Marimastat reduced the activation of JNK, ERK1/2, AKT, and STAT3 in AATF control cells. In contrast, Marimastat had no effect on AATF knockdown QGY‐7703 cells (Fig. 7D). Taken together, these findings provide supporting evidence that inhibition of TACE activity prevents the cleavage and subsequent release of TNF‐α from the Kupffer cells. This further leads to the inhibition of hepatic AATF and associated downstream signaling pathways, preventing the development and progression of liver tumorigenesis (Fig. 8).

## 4 Discussion

MASH‐HCC refers to the development of HCC in individuals with pre‐existing metabolic dysfunction‐associated steatohepatitis (MASH). It is an increasingly recognized complication of MASH and is becoming a leading cause of liver cancer worldwide [4]. It is important to note that MASLD (formerly known as NAFLD) is a term that has emerged recently to highlight the strong association between fatty liver disease and metabolic disorders. MASLD is closely associated with metabolic disorders such as obesity, insulin resistance, type 2 diabetes, dyslipidemia, and hypertension [34]. These risk factors contribute to the accumulation of fat in the liver, which can lead to inflammation and liver cell injury and potentially progress to more severe forms of liver disease, such as steatohepatitis (MASH), fibrosis, cirrhosis, and even liver cancer. Thus, the pathogenesis of MASH‐associated HCC encompasses an intricate terrain consisting of various mechanisms pertaining to oxidative stress, ER stress, immune and inflammatory responses, DNA damage, and autophagy [35]. Unlike other types of liver cancer, such as viral hepatitis‐associated HCC, there are currently no established biomarkers or screening tests specifically designed for MASH‐HCC. This slow and insidious progression makes it challenging to identify the transition points and detect HCC at an early stage. Despite significant advancements in HCC management and the availability of multiple treatment options, MASH‐HCC can still go undiagnosed in its early stages or be deemed inappropriate for curative therapies due to the absence of cirrhosis or the presence of high‐risk comorbidities [36, 37]. These challenges mark the need for the identification of key molecular drivers of HCC in MASH to develop an effective therapeutic regime.

Keeping that objective insight, we had previously investigated the regulatory role of AATF in MASH‐HCC and found that hepatic AATF was upregulated in the diet‐induced animal model of non‐alcoholic fatty liver disease (DIAMOND) [16]. By surmounting the limitations and hurdles associated with employing a mouse model for MASH‐HCC, DIAMOND mice exhibited a remarkable resemblance to human pathophysiology, with the exception of the time required for liver tumor formation, which averaged around 52 weeks. Henceforth, in the present study, we used a FAT (Fibrosis And Tumors)‐MASH murine model fed a western‐style diet (high‐fat, 21%; high sucrose, 41%; high cholesterol, 1.25%) and sugar water (18.9 g·L^−1^ d‐glucose and 23.1 g·L^−1^ d‐fructose) along with CCl_4_ (very low dose‐0.2 μL·g^−1^, once per week) as a fibrosis accelerator [24]. Mice rapidly develop severe MASH after 12 weeks with fibrosis and subsequent HCC at 24 weeks. Our study findings are significantly enhanced by the metabolic, histological, and transcriptomic characteristics observed in these mice, which closely resemble those found in human MASH‐HCC. This similarity boosts the translational prospects of our research. Consistent with our previous studies, we also observed upregulated AATF expression in this preclinical model. WD/CCl_4_ mice showed elevated levels of serum liver enzymes and cholesterol, significant steatosis, hepatocellular ballooning, and lobular inflammation at 12 weeks, and advanced fibrosis and tumor development at 24 weeks. Besides, inflammatory, ER stress, and fibrogenic markers were also upregulated, supporting the progression of MASH to HCC. Additionally, the confirmation of elevated levels of stress kinases (ERK1/2 and JNK), proliferative marker (AKT), and hepato‐oncogenic gene (STAT3) underscores the significance of metabolic reprogramming in driving oncogenesis in the milieu of chronic inflammation.

In the context of MASH, TNF‐α is known to be involved in the progression of the disease, and increased levels of TNF‐α have been observed in individuals with MASH [38]. TNF‐α is produced by various cells, including immune cells, and it promotes inflammation by activating other immune cells and promoting the release of inflammatory cytokines [39]. The biologically active form of TNF‐α, which is the soluble TNF‐α, is the resultant of the cleavage of pro TNF‐α protein on the cell membrane by TACE, an enzyme also referred to as ADAM17 [13]. Thereby inhibiting TACE activity can reduce the release of TNF‐α, which plays a detrimental role in inflammatory diseases, including MASH. Of note, we have previously determined that the proinflammatory cytokine, TNF‐α via SREBP1 upregulates AATF expression, which acts as a potential driver of HCC in MASLD [16]. Considering that there are no pharmacological inhibitors specific to AATF, we hypothesized whether targeting an upstream molecule such as TNF‐α could offer a potential therapeutic strategy to prevent AATF‐mediated MASH‐HCC. However, taking into account the limitations or drawbacks associated with the use of TNF‐α inhibitors, such as increased susceptibility to infections, injection site reactions, cost, and availability, we employed an inhibitor of TACE instead. Similarly, dysregulation of TACE activity has been reported in several diseases, including MASH and cancer [40]. Studies have shown that increased TACE activity induces insulin resistance and steatosis, and inhibition of the enzyme reverses hepatic steatosis in mouse models [14, 15]. Matsui et al. [41] showed that high‐fat diet‐induced overexpression of TACE induces adipose tissue inflammation along with fibrosis. Implications of increased TACE activity have been reported in atherosclerosis, diabetes, adipose tissue, and liver metabolism [14, 42, 43, 44]. Thus, it became evident that TACE could be an attractive candidate to explore its possible role in AATF‐mediated metabolic inflammation to cancer.

Interestingly, TACE activity is controlled by TIMP3, an endogenous inhibitor of TACE, and thus, TIMP3/TACE modulation is commonly observed in insulin resistance, diabetes, inflammation, and tumor angiogenesis [44, 45, 46]. Studies have reported that the deficiency of TIMP3 induces hepatic steatosis and adipose tissue inflammation in mice [45]. In the present study, WD/CCl_4_ mice showed increased TACE activity, and as expected, low levels of TIMP3 were observed. The downregulation of TIMP3 is attributed to SIRT1, which is also downregulated in MASH. Studies have shown a functional link between SIRT1 and TIMP3, where SIRT1 increases the promoter activity of TIMP3 [30]. Thus, our study provides proof of concept of the involvement of the SIRT1/TIMP3/TACE axis in promoting the release of TNF‐α leading to the upregulation of AATF, a key molecular driver of MASH‐HCC. Studies by de Meijer et al., have provided evidence that Marimastat, a pharmacological TACE inhibitor, would ameliorate steatosis and inflammation following hepatectomy or repeated hepatoxin‐induced liver injury [15, 47]. Consistent with these data, Marimastat treatment in WD/CCl_4_ mice markedly reduced hepatic injury, insulin resistance, inflammation, fibrosis, and tumor burden. However, Marimastat, a TACE inhibitor, is known to exhibit inhibitory effects against a wide range of matrix metalloproteinase (MMPs), including MMP‐1, MMP‐2, MMP‐3, MMP‐7, MMP‐9, and MMP‐13 [48]. Notably, this broad‐spectrum activity allows it to potentially target multiple pathways involved in tissue remodeling and cancer progression. Despite the limitations of generalizing our findings to Marimastat and acknowledging the potential influence of Marimastat's off‐target effects on our findings, it is probable that prolonged use of inhibitors with anti‐TACE activity will yield similar divergent effects on liver injury and fibrosis.

Moreover, the current study offers a deeper understanding of how TACE inhibition effectively thwarts the AATF‐mediated progression of MASH‐HCC, shedding light on the underlying mechanisms involved. The *in vivo* data were corroborated with the *in vitro* studies wherein human HCC cells, QGY‐7703 cells, which expressed high levels of AATF, showed reduced AATF expression and decreased levels of TNF‐α and TACE activity upon Marimastat treatment. Consistent with these data, the mRNA levels of IL‐1β, IL‐6, CHOP, Grp78, CD31, and AFP were significantly reduced with the treatment of Marimastat. In oncogenic signaling, activation of JNK (c‐Jun N‐terminal kinase), ERK1/2 (extracellular signal‐regulated kinase 1/2), and AKT (protein kinase B) plays a significant role in contributing to tumor development and progression [35]. The most compelling proof of TACE activity's involvement in MASH‐HCC, mediated by AATF, lies in the observation that Marimastat, a TACE inhibitor, reduces JNK, ERK1/2, and AKT activation in AATF control cells. Conversely, it had no effect on AATF knockdown QGY‐7703 cells.

## 5 Conclusion

This study contributes to the expanding pool of evidence that reinforces the crucial involvement of TACE and TNF‐α in the transition from MASH to HCC, mediated by AATF, underscoring the potential of TACE as a therapeutic target to impede tumorigenesis. Moreover, the results establish a robust groundwork for conducting extensive exploration of TACE‐specific pharmacologic inhibitors in human trials, with the primary goal of alleviating the burden of HCC attributed to MASH. Furthermore, we advocate further investigations that focus on liver‐specific targeting of AATF or the generation of liver‐specific AATF knockout mice, as these approaches will facilitate a comprehensive understanding of the impact of AATF throughout different stages of MASLD.

## Conflict of interest

The authors declare no conflict of interest.

## Author contributions

ANS, DS, and DPK were involved in conceptualization, methodology and data analysis. SS and DPK performed pathological evaluation of liver tissues. PMV and PKS provided necessary material and helped in analysis and interpretation of data. SK intellectually supported the design of the study and data analysis. ANS and DPK prepared the manuscript. DPK acquired funding and supervised the research. PKS and SK also critically reviewed the manuscript. All authors read and approved the final version of the manuscript.

## Supporting information


**Fig. S1.** Evaluation of metabolic parameters and histology in mice fed with CD/CCl4 or WD/CCl4.
**Fig. S2.** TCGA analysis of TNF‐α converting enzyme (TACE) and apoptosis antagonizing transcription factor (AATF) expression.
**Fig. S3.** Serum tests for liver injury and histological features in mice treated with Marimastat.
**Fig. S4.** (A) TACE activity in QGY‐7703 cells upon Marimastat treatment. Human hepatocellular carcinoma (HCC) cells, QGY‐7703, were treated with different concentrations of Marimastat (0, 10, 50, 100, 500, or 1000 nm), and TNF‐α converting enzyme (TACE) activity was measured. Data expressed as mean ± SEM. **P < 0.001 or *P < 0.05 compared with untreated control. (B) TACE activity and (C) TNF‐α were also measured in Hep3B cells treated with or without Marimastat.
**Table S1.** List of primer sequences used in the study.

## Data Availability

All data associated with this study are included in this article and its associated additional information files.
