# Supplementary material for: TACE inhibition: a promising therapeutic intervention against AATF‐mediated steatohepatitis to hepatocarcinogenesis
Source: Mol Oncol. 2024 Apr 1;18(8):1940–57. doi: 10.1002/1878-0261.13646 (PMC11306524; doi:10.1002/1878-0261.13646)
Supplement: Supplementary file 1 — Fig. S1. Evaluation of metabolic parameters and histology in mice fed with CD/CCl4 or WD/CCl4. Fig. S2. TCGA analysis of TNF‐α converting enzyme (TACE) and apoptosis antagonizing transcription factor (AATF) expression. Fig. S3. Serum tests for liver injury and histological features in mice treated with Marimastat. Fig. S4. (A) TACE activity in QGY‐7703 cells upon Marimastat treatment. Human hepatocellular carcinoma (HCC) cells, QGY‐7703, were treated with different concentrations of Marimastat (0, 10, 50, 100, 500, or 1000 nm), and TNF‐α converting enzyme (TACE) activity was measured. Data expressed as mean ± SEM. **P < 0.001 or *P < 0.05 compared with untreated control. (B) TACE activity and (C) TNF‐α were also measured in Hep3B cells treated with or without Marimastat. Table S1. List of primer sequences used in the study. [file MOL2-18-1940-s001.zip › Supporting Information-Figure Legends.pdf]

Supporting Information:

Supplementary Figure 1. Evaluation of metabolic parameters and histology in mice fed with CD/CCl_4 or WD/CCl_4. (A) liver weight, (B) serum cholesterol, (C) fasting glucose, and (D) fasting insulin were measured. Histology scores for (E) steatosis, (F) lobular inflammation, and (G) hepatocyte ballooning was determined. Statistical significance was analyzed by a student's t-test. Data expressed as mean±SEM for n=6 mice per group. ** $p < 0.001$ or * $p < 0.05$ compared to CD/CCl_4.

Supplementary Figure 2. TCGA analysis of TACE and AATF expression. (A) AATF, (B) TACE expression in human HCC samples compared to adjacent normal. (C) gene expression correlation of TACE and AATF in human HCC samples.

Supplementary Figure 3. Serum tests for liver injury and histological features in mice treated with Marimastat. Mice were fed with CD/CCl_4 or WD/CCl_4 for 12 weeks and then treated with vehicle control or Marimastat for an additional 12 weeks. (A) body weight, (B) liver weight, (C) Calorie intake, (D) serum cholesterol, (E) fasting glucose, and (F) fasting insulin were measured. Histology scores for (G) steatosis, (H) lobular inflammation, and (I) hepatocyte ballooning was determined. Statistical significance was analyzed by a student's t-test. Data expressed as mean±SEM for n=6 mice per group. ** $p < 0.001$ or * $p < 0.05$ compared to CD/CCl_4 vehicle control; ## $p < 0.001$ or # $p < 0.05$ compared to WD/CCl_4 vehicle control.

Supplementary Figure 4. (A) TACE activity in QGY-7703 cells upon Marimastat treatment. Human HCC cells, QGY-7703, were treated with different concentrations of Marimastat (0, 10, 50, 100, 500, or 1000 nM), and TACE activity was measured. Data expressed as mean±SEM. **$p < 0.001$ or *$p < 0.05$ compared to untreated control. (B) TACE activity and (C) TNF-α were also measured in Hep3B cells treated with or without Marimastat. Statistical significance was analyzed by a student's t-test. Data expressed as mean±SEM for n=3 independent experiments. ## $p < 0.001$ or # $p < 0.05$ compared to Hep3B cells vehicle control.

Supplementary Table 1: List of primer sequences used in the study.
