# Supplementary figures and images for: TACE inhibition: a promising therapeutic intervention against AATF‐mediated steatohepatitis to hepatocarcinogenesis
Source: Mol Oncol. 2024 Apr 1;18(8):1940–57. doi: 10.1002/1878-0261.13646 (PMC11306524; doi:10.1002/1878-0261.13646)

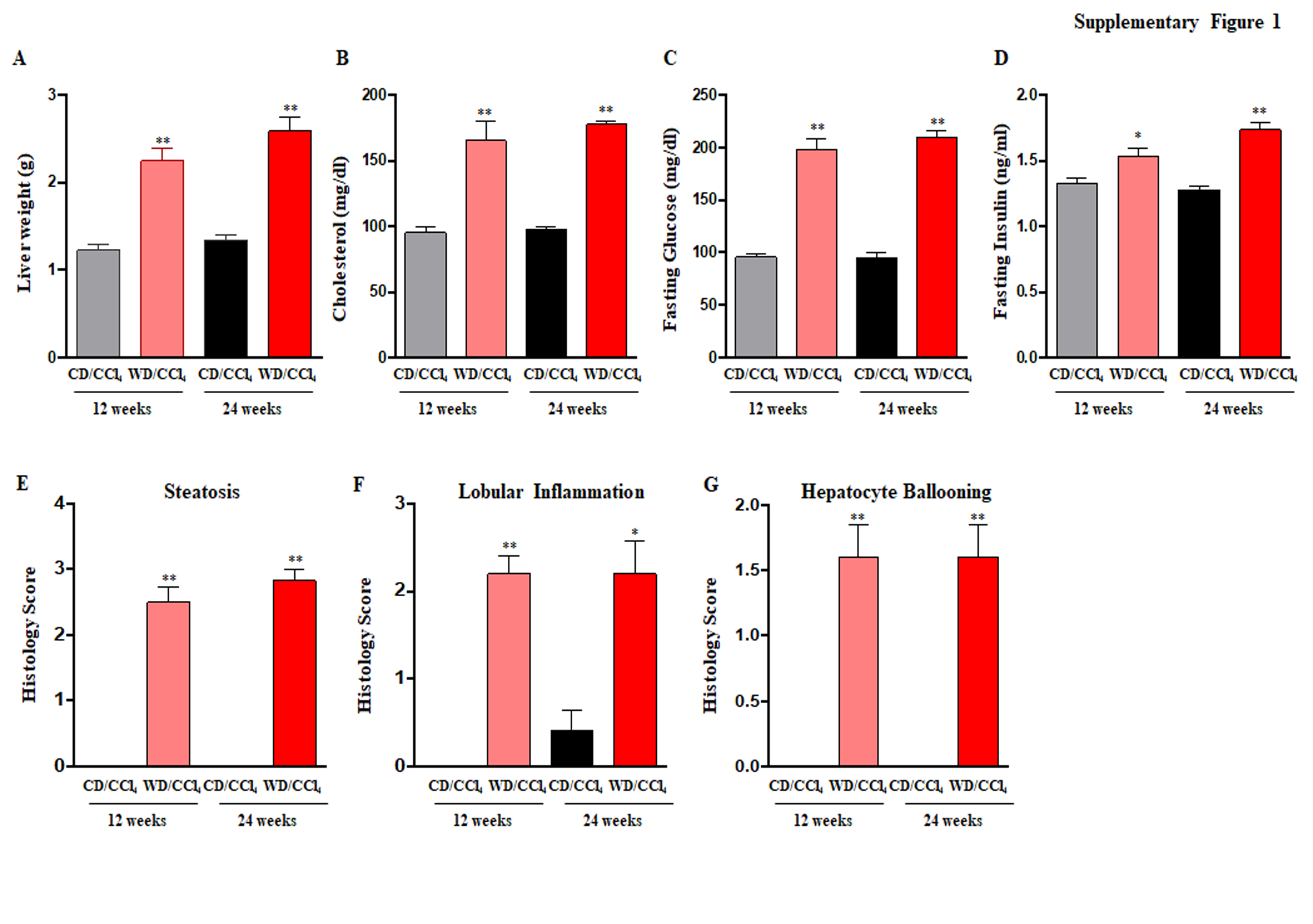

Supplement: Supplementary file 1 — Fig. S1. Evaluation of metabolic parameters and histology in mice fed with CD/CCl4 or WD/CCl4. Fig. S2. TCGA analysis of TNF‐α converting enzyme (TACE) and apoptosis antagonizing transcription factor (AATF) expression. Fig. S3. Serum tests for liver injury and histological features in mice treated with Marimastat. Fig. S4. (A) TACE activity in QGY‐7703 cells upon Marimastat treatment. Human hepatocellular carcinoma (HCC) cells, QGY‐7703, were treated with different concentrations of Marimastat (0, 10, 50, 100, 500, or 1000 nm), and TNF‐α converting enzyme (TACE) activity was measured. Data expressed as mean ± SEM. **P < 0.001 or *P < 0.05 compared with untreated control. (B) TACE activity and (C) TNF‐α were also measured in Hep3B cells treated with or without Marimastat. Table S1. List of primer sequences used in the study. [file MOL2-18-1940-s001.zip › Supplementary Figure 1.tif]

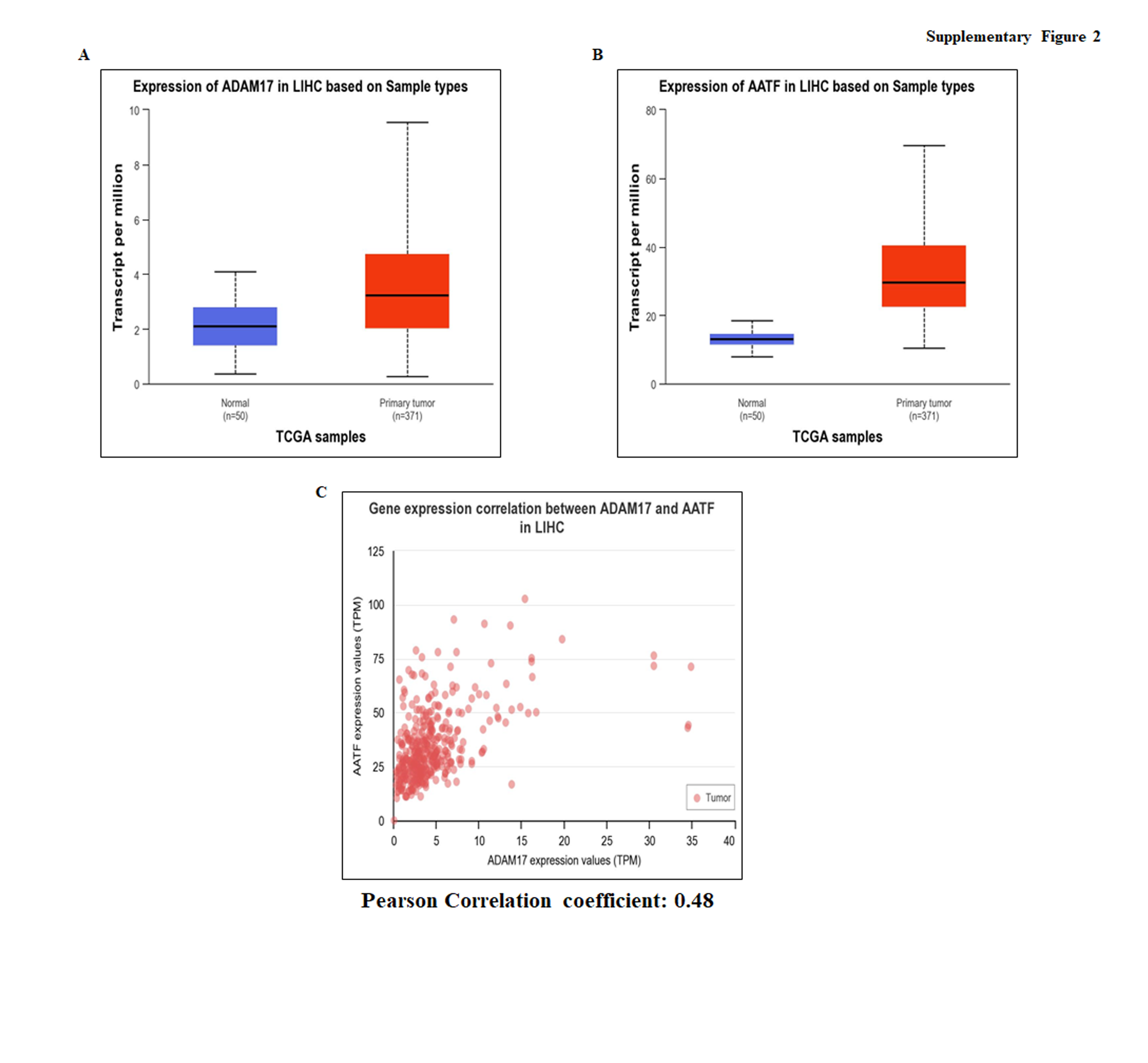

Supplement: Supplementary file 1 — Fig. S1. Evaluation of metabolic parameters and histology in mice fed with CD/CCl4 or WD/CCl4. Fig. S2. TCGA analysis of TNF‐α converting enzyme (TACE) and apoptosis antagonizing transcription factor (AATF) expression. Fig. S3. Serum tests for liver injury and histological features in mice treated with Marimastat. Fig. S4. (A) TACE activity in QGY‐7703 cells upon Marimastat treatment. Human hepatocellular carcinoma (HCC) cells, QGY‐7703, were treated with different concentrations of Marimastat (0, 10, 50, 100, 500, or 1000 nm), and TNF‐α converting enzyme (TACE) activity was measured. Data expressed as mean ± SEM. **P < 0.001 or *P < 0.05 compared with untreated control. (B) TACE activity and (C) TNF‐α were also measured in Hep3B cells treated with or without Marimastat. Table S1. List of primer sequences used in the study. [file MOL2-18-1940-s001.zip › Supplementary Figure 2.tif]

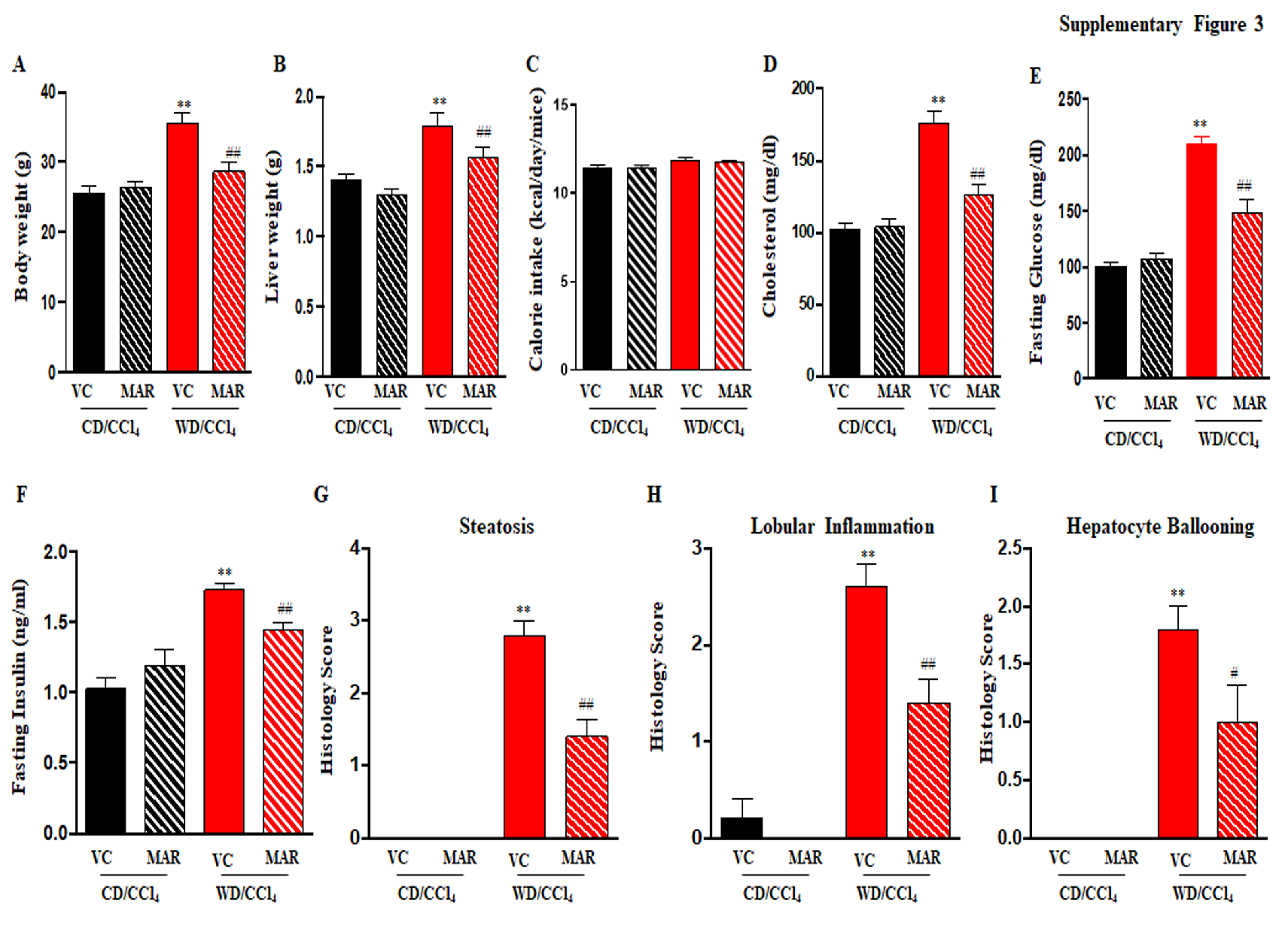

Supplement: Supplementary file 1 — Fig. S1. Evaluation of metabolic parameters and histology in mice fed with CD/CCl4 or WD/CCl4. Fig. S2. TCGA analysis of TNF‐α converting enzyme (TACE) and apoptosis antagonizing transcription factor (AATF) expression. Fig. S3. Serum tests for liver injury and histological features in mice treated with Marimastat. Fig. S4. (A) TACE activity in QGY‐7703 cells upon Marimastat treatment. Human hepatocellular carcinoma (HCC) cells, QGY‐7703, were treated with different concentrations of Marimastat (0, 10, 50, 100, 500, or 1000 nm), and TNF‐α converting enzyme (TACE) activity was measured. Data expressed as mean ± SEM. **P < 0.001 or *P < 0.05 compared with untreated control. (B) TACE activity and (C) TNF‐α were also measured in Hep3B cells treated with or without Marimastat. Table S1. List of primer sequences used in the study. [file MOL2-18-1940-s001.zip › Supplementary Figure 3.tif]

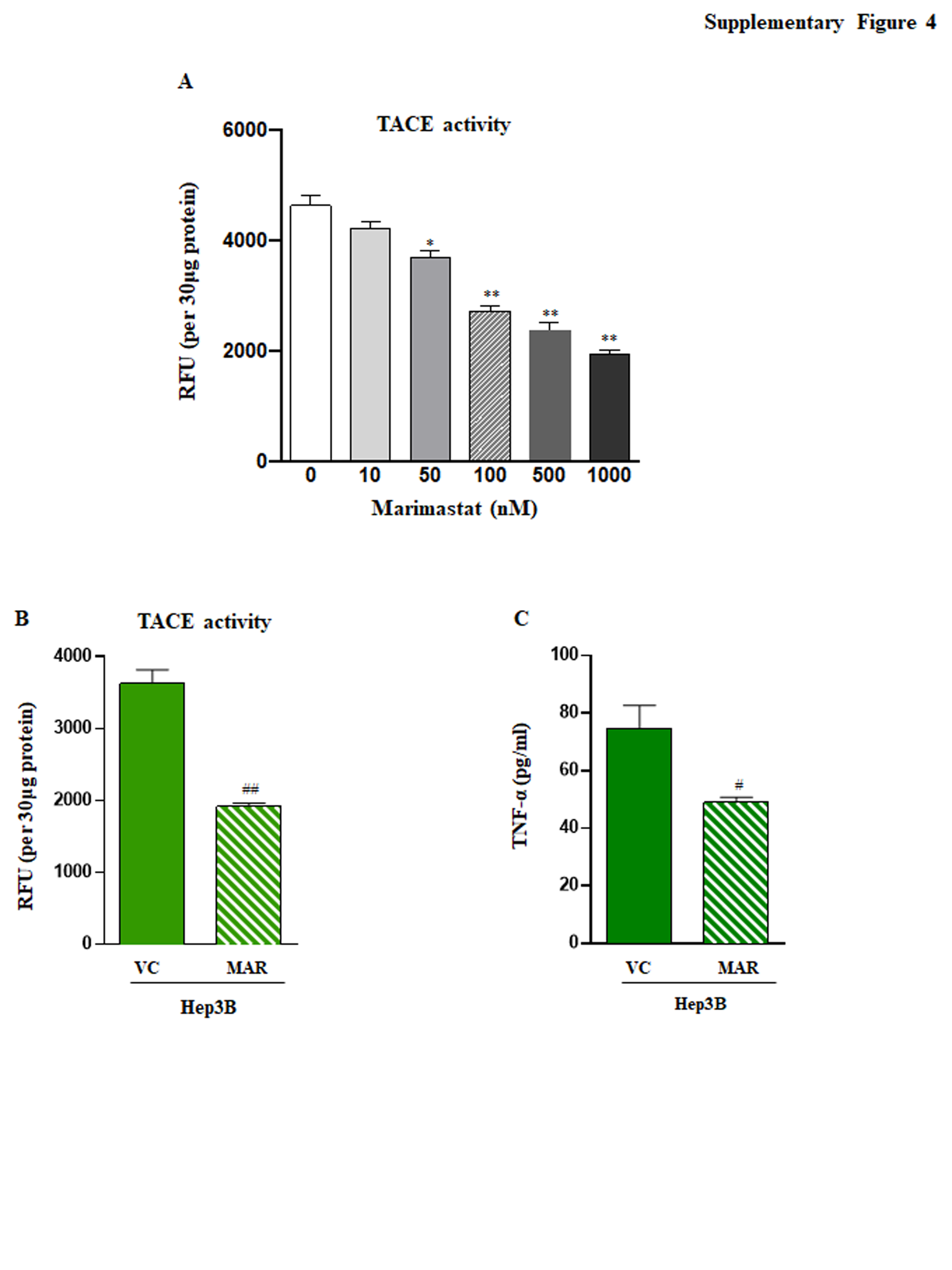

Supplement: Supplementary file 1 — Fig. S1. Evaluation of metabolic parameters and histology in mice fed with CD/CCl4 or WD/CCl4. Fig. S2. TCGA analysis of TNF‐α converting enzyme (TACE) and apoptosis antagonizing transcription factor (AATF) expression. Fig. S3. Serum tests for liver injury and histological features in mice treated with Marimastat. Fig. S4. (A) TACE activity in QGY‐7703 cells upon Marimastat treatment. Human hepatocellular carcinoma (HCC) cells, QGY‐7703, were treated with different concentrations of Marimastat (0, 10, 50, 100, 500, or 1000 nm), and TNF‐α converting enzyme (TACE) activity was measured. Data expressed as mean ± SEM. **P < 0.001 or *P < 0.05 compared with untreated control. (B) TACE activity and (C) TNF‐α were also measured in Hep3B cells treated with or without Marimastat. Table S1. List of primer sequences used in the study. [file MOL2-18-1940-s001.zip › Supplementary Figure 4.tif]
